# Exploring Tb^3+^-Mediated Interactions with Glutathione-Capped Gold Nanoclusters to Develop a Fluorophore-Modified Ratiometric Probe toward Lactoferrin

**DOI:** 10.1021/acs.jpcb.5c06319

**Published:** 2025-10-30

**Authors:** Chun-Hsin Kuo, Bo-Yu Liu, Shin-Yi Feng, Cheng-Kang Chiang, Ming-Mu Hsieh, Wei-Lung Tseng

**Affiliations:** † Department of Chemistry, 34874 National Sun Yat-Sen University, No. 70 Lienhai Rd., Kaohsiung 80424, Taiwan; ‡ Department of Chemistry, 63373 National Dong Hwa University, Shoufeng, Hualien 974301, Taiwan; § Department of Chemistry, National Kaohsiung Normal University, No.62, Shenjhong Rd., Yanchao District, Kaohsiung City 82446, Taiwan; ∥ Center for Nanoscience & Nanotechnology, National Sun Yat-Sen University, No. 70 Lienhai Rd., Kaohsiung 80424, Taiwan; ⊥ School of Pharmacy, College of Pharmacy, Kaohsiung Medical University, No.100, Shiquan 1st Rd., Kaohsiung 80708, Taiwan

## Abstract

A ratiometric fluorescent probe for lactoferrin (Lf) was developed by conjugating fluorescent BDP-FL molecules onto glutathione (GSH)-capped gold nanoclusters, Au_29–43_(GSH)_27–37_, followed by terbium ion (Tb^3+^)-induced aggregation-induced emission enhancement (AIEE) of the clusters. The resultant BDP-FL-conjugated AIEE dots exhibit characteristic emissions at 517 and 606 nm. Conjugation of BDP-FL to the Au_29–43_(GSH)_27–37_ clusters provides a green-emissive internal reference, enabling ratiometric signal output. Upon addition of Lf, competitive binding with Tb^3+^ disrupts the aggregates, leading to selective attenuation of the red emission from Au_29–43_(GSH)_27–37_ while preserving the green BDP-FL emission. This ratiometric design affords a wide linear range (0.01–4.0 mg/mL), a low limit of detection (3.4 μg/mL), and excellent reproducibility (relative standard deviation < 1.2%). Importantly, the probe remains effective in 10-fold-diluted human tear samples, achieving recovery rates of 99.98–101.8% and providing results consistent with capillary electrophoresis. Mechanistic studies reveal that Tb^3+^ reduces electrostatic repulsion and enhances van der Waals and bridging interactions, thereby promoting aggregation of Au_29–43_(GSH)_27–37_. Using the Derjaguin–Landau–Verwey–Overbeek theoretical model, the relative contributions of van der Waals, electrostatic, and bridging interaction energies were quantified, offering deeper insight into the aggregation mechanism. This ratiometric AIEE probe demonstrates practicality, sensitivity, and reliability for Lf determination in clinical samples and may provide guidance for the rational design of nanomaterials with tailored optical properties.

## Graphical abstract

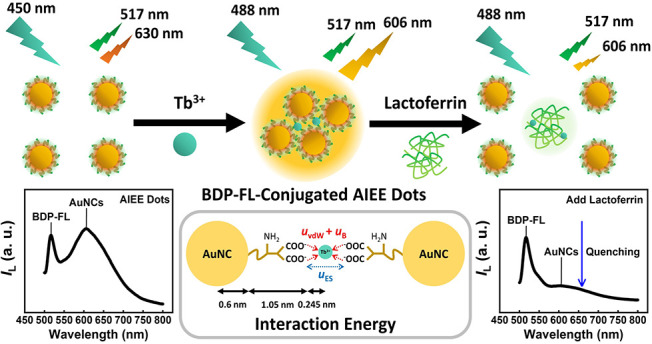

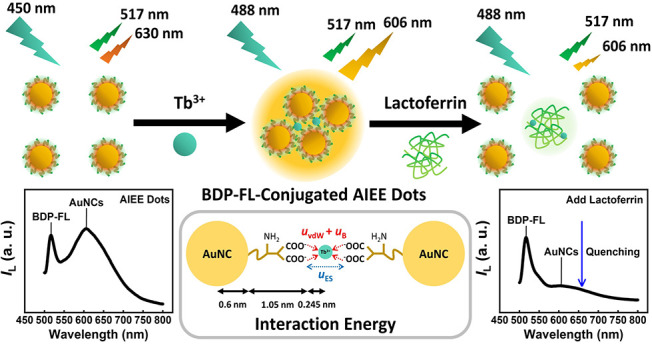

## 1 Introduction

Lactoferrin (Lf), with a molecular weight of approximately 80 kDa, is a member of the transferrin family and is widely present in various human secretions, including feces, milk, serum, and tears. Lf exhibits multiple biological functions, including antibacterial, antiviral,^1^ antioxidant, and anti-inflammatory properties,^2^ which make it a potential diagnostic marker for several diseases such as inflammatory bowel disease,^3^ Alzheimer’s disease,^4^ and dry eye disease (DED).^5–7^ DED can cause visual impairment and damage to the ocular surface. Because Lf possesses antibacterial and anti-inflammatory biological functions, it can scavenge reactive oxygen and hydrocarbon radicals from tears. However, DED patients have lower levels of Lf in their tears, making their eyes more susceptible to oxidative metabolites.^8^ They secrete fewer tears than healthy individuals, mainly because tear volume is positively correlated with the Lf concentration.^9^ With the growing prevalence of DED driven by prolonged digital screen exposure, there is an urgent need to develop rapid and straightforward methods for Lf detection.

Currently, several methods are available for determining Lf, including high-performance liquid chromatography,^10,11^ capillary electrophoresis,^12,13^ radial immunodiffusion,^14^ enzyme-linked immunosorbent assay,^15^ electrochemical sensors,^16^ surface plasmon resonance (SPR),^17^ and fluorescent sensors.^18^ Among these techniques, fluorescent sensors stand out for their ease of operation, low cost, high sensitivity, and excellent performance, which have motivated the development of sensors incorporating aptamers,^19–21^ carbon-related quantum dots,^22,23^ terbium ions (Tb^3+^),^24,25^ and molecularly imprinted polymers.^26^ As an example of tear Lf detection, Zhang et al. reported a fluorescence polarization- and fluorescence resonance energy transfer (FRET)-based aptasensor, consisting of Lf aptamer-conjugated carbon dots and graphene oxide nanosheets, for the detection Lf in tear samples.^19,20^ Yamada et al. developed a microfluidic paper-based analytical device in which the emission band of the Lf-Tb^3+^ complex along the paper strip correlates with the Lf concentration.^24^ Similarly, Tsai et al. introduced a portable device for luminescent detection of tear Lf based on Lf-induced luminescence enhancement of Tb^3+^.^25^ This device effectively distinguishes the lower tear Lf levels in patients with Sjögren’s syndrome dry eye (∼0.087 mg/mL) and non-Sjögren dry eye (∼0.337 mg/mL) from those in healthy individuals (∼1.27 mg/mL). Although these luminescent sensors exhibit excellent sensitivity and selectivity toward Lf, their practical applications are often limited by poor signal reproducibility. This issue mainly arises from fluctuations in the excitation light source, changes in the concentration of the fluorescent probe, and environmental interference (such as variations in temperature, ionic strength, and solution pH).^27^

In response to these limitations, a ratiometric sensing probe is introduced as a promising alternative. By measuring the ratio of two signals emitted by the probe (one responsive to the analyte and the other serving as an internal reference), ratiometric sensors can effectively correct for external interference and instrument variability. Since exhibiting red-light emission,^28^ easily functionalizable nature, metal ion-mediated aggregation-induced emission enhancement (AIEE),^29^ and large Stokes shifts,^30^ gold nanoclusters (AuNCs) are highly suitable for use as ratiometric probes for biomolecules.^31,32^ These features inspired us to develop a ratiometric sensing platform for accurate, precise, and reproducible detection of Lf in tear samples. Considering the specific interaction between Tb^3+^ and Lf,^24,25^ we introduced Tb^3+^ to induce aggregation-induced emission enhancement (AIEE) of glutathione-capped AuNCs (GSH–AuNCs) through coordination between the carboxyl groups of GSH and Tb^3+^. Furthermore, green-emitting BDP-FL molecules were conjugated to the carboxyl groups of the capped GSH because their emission peak scarcely overlaps with that of the AuNCs. This dual-emission design enables ratiometric signal output, where the emission from AuNC-Tb^3+^ aggregates responds to Lf binding events, while the BDP–FL emission serves as an internal reference (Figure 1). In addition, we elucidate the AIEE mechanism according to the Derjaguin–Landau–Verwey–Overbeek (DLVO) theory, revealing that Tb^3+^ coordinates with the carboxylic groups of GSH to reduce electrostatic repulsion, thereby promoting AuNC aggregation and enhancing emission. This strategy combines the recognition ability of Tb^3+^ with the ratiometric advantage of AuNC-based dual-emission probes, demonstrating its practical applicability for Lf detection in tear samples.

**Figure 1:**
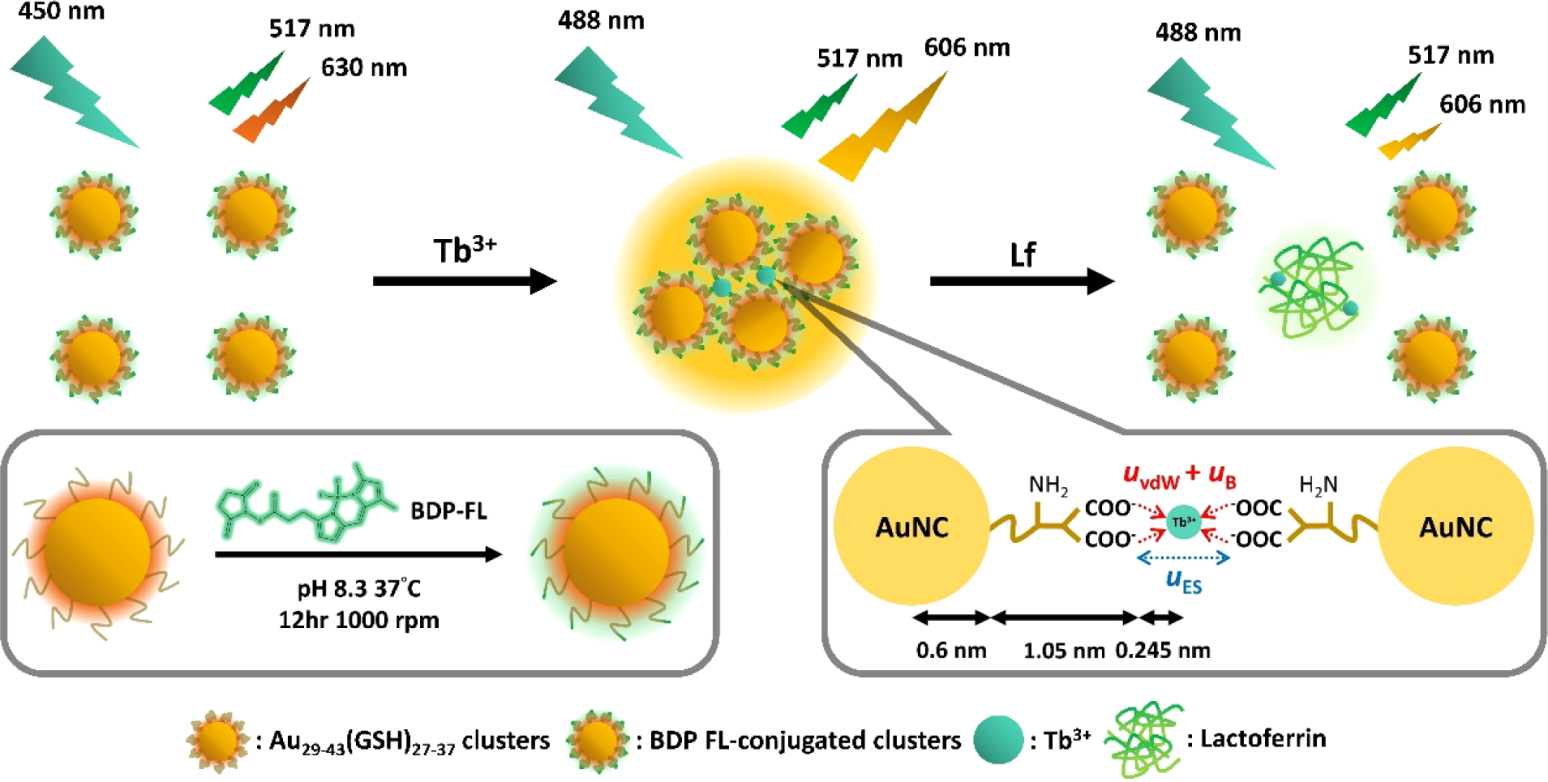
Schematic illustrating fabrication of a ratiometric probe via Tb^3+^-induced aggregation of BDP-FL-conjugated Au_29–43_(GSH)_27–37_ clusters, and its application to ratiometric detection of Lf. To ensure that BDP-FL and the AIEE dots exhibit similar peak fluorescence intensities, an excitation wavelength of 488 nm was selected in the sensing system.

## 2 Experimental Section

### 2.1 Preparation of GSH-AuNCs and Tb^3+^-related AIEE Dots

The synthesis of Au_29–43_(GSH)_27–37_ was performed with slight modifications to a previously reported protocol.^33^ A solution consisting of HAuCl_4_ (1 mL, 0.2 M), GSH (0.4 mL, 0.1 M), and deionized water (8.6 mL) was shaken at 100 rpm for 30 min. After that, excess GSH (2 mL, 0.2 M) was introduced to 7 mL of the above mixture, followed by drying in an oven at 70 °C for 24 h. The dried product was purified by dialysis (molecular weight cutoff: 1 kDa) to yield Au_29–43_(GSH)_27–37_. The concentration of gold (*C*_Au_) in Au_29–43_(GSH)_27–37_ was measured to be 1.74 mg/mL by atomic absorption spectroscopy (AAnalyst 200, PerkinElmer-SCIEX, Thornhill, ON, Canada). Tb^3+^-related AIEE dots were prepared by mixing 50 μL of Au_29–43_(GSH)_27–37_, 100 μL of 100 mM HEPES buffer (pH 7.0), 750 μL of deionized water, and 100 μL of Tb^3+^ solution at different concentrations, followed by gentle shaking at room temperature for 10 min.

### 2.2 Synthesis of BDP-FL-Conjugated Clusters and BDP-FL-Conjugated AIEE Dots

A mixture of Au_29–43_(GSH)_27–37_ (300 μL, *C*_Au_ = 22.2 mg/mL), HEPES buffer (200 μL, 100 mM; pH 8.3), and BDP-FL solution (25 μL, 0.1 mg/mL) was shaken at 37 °C and 1000 rpm for 12 h. Subsequently, the resultant solution was purified using a centrifugal filter unit (molecular weight cutoff: 1 kDa) to obtain BDP-FL-conjugated clusters. BDP-FL-conjugated AIEE dots were then prepared by mixing BDP-FL-conjugated clusters (50 μL, *C*_Au_ = 9.89 mg/mL), deionized water (850 μL), and Tb^3+^ solution (100 μL, 0–0.01 M), followed by gentle shaking at ambient temperature for 10 min.

### 2.3 Determination of the Dissociation Constant

The dissociation constant (*K*_d_) was determined by fitting the binding data to the Hill equation, as shown below:

$$\frac{\mathrm{Bound}‐\mathrm{ligand concentration}}{\mathrm{Maximum binding capacity}}=\theta =\frac{{[L]}^{n}}{Kd^{n}+{[L]}^{n}}$$

where θ is the fractional binding, [L] is the free ligand concentration, and *n* is the Hill coefficient describing cooperativity. Since θ can be inferred from the change in fluorescence intensity, the equation can be rewritten as

1 $$\frac{I_{L}-I_{0}}{I_{\max}-I_{0}}=\theta =\frac{{[L]}^{n}}{Kd^{n}+{[L]}^{n}}$$

where *I*_0_ is the luminescence intensity in the absence of ligand (baseline), *I*_max_ is the intensity at saturating ligand concentration, and *I*_L_ is the intensity at an intermediate ligand concentration. By recording *I*_L_ at a series of ligand concentrations and performing nonlinear regression of eq 1, values of *K*_d_ and *n* are extracted.

### 2.4 Sensing of Lf

Aliquots of Tb^3+^-related AIEE dots (300 μL, *C*_Au_ = 0.087 mg/mL) were incubated with a solution of Lf (100 μL, 0.01–3.0 mg/mL) at ambient temperature for 10 min. The luminescence spectra of the resultant solution were recorded at an excitation wavelength of 450 nm. A calibration curve was constructed by plotting the luminescence intensity at 607 nm against the Lf concentration; each data point represented three independent measurements. For the ratiometric probe, a solution of BDP-FL-conjugated AIEE dots (300 μL, *C*_Au_ = 0.49 mg/mL, chosen to ensure sufficient fluorescence intensity of BDP-FL for the ratiometric measurement) was incubated with different concentrations of Lf (100 μL, 0.01–4.0 mg/mL) at ambient temperature for 10 min, followed by spectral collection at an excitation wavelength of 488 nm. It is noted that diluting the gold concentration to match that used in Figure 4 would result in BDP-FL fluorescence signals too weak to support reliable ratiometric measurements. Therefore, the concentration of gold concentration in the ratiometric probe was adjusted to be 0.49 mg/mL. The *I*_BDP_/*I*_AuNCs_ value was plotted against the Lf concentration to generate the ratiometric calibration curve. The average value and error bar for each data point were obtained from three independent measurements.

Tear samples were collected from the canthus of a healthy 21 year-old male volunteer using a dropper. The collection process followed the ethical guidelines of the World Medical Association’s 1975 Declaration of Helsinki and was approved by the Institutional Review Board of the National Cheng Kung University Human Research Ethics Committee (NCKU HREC-E111-607-2). The collected tears were diluted 10-fold and filtered through a 0.22 μm nitrocellulose membrane to remove impurities. Subsequently, we spiked the diluted tear samples with different concentrations of standard Lf (0.05–4.0 mg/mL). The subsequent steps were the same as those described above for Lf sensing.

## 3 Results and Discussion

### 3.1 Tb^3+^-Induced AIEE of GSH-AuNCs

Xie’s group reported a NaBH_4_- and carbon monoxide-free method for synthesizing GSH–AuNCs with a high thiolate-to-gold ratio (>0.85),^33^ in contrast to the well-known Au_25_(GSH)_18_ and Au_18_(GSH)_14_ clusters.^30^ In this approach, GSH functions not only as a capping ligand but also as a reducing agent. Based on slab-gel electrophoresis and electrospray ionization mass spectrometry, the resulting clusters consisting of Au_29_(GSH)_27_, Au_30_(GSH)_28_, Au_36_(GSH)_32_, Au_39_(GSH)_35_, and Au_43_(GSH)_37_ were collectively designated as Au_29–43_(GSH)_27–37_ clusters.^33,34^ Our group has previously demonstrated that the Au_29–43_(GSH)_27–37_ clusters possess the AIEE properties in the presence of cerium ions,^29^ positively charged peptides,^35^ and surfen.^36^ It is suggested that a high thiol-to-gold ratio in the Au_29–43_(GSH)_27–37_ clusters facilitates the complexation with AIEE trigger molecules through a relatively high number of functional groups compared to the Au_25_(GSH)_18_ and Au_18_(GSH)_14_ clusters. Inspired by these findings, we sought to investigate whether Tb^3+^ could also drive the AIEE of the Au_29–43_(GSH)_27–37_ clusters. Prior to this investigation, we verified that the synthesis procedure described in the experimental section yielded the Au_29–43_(GSH)_27–37_ clusters. The as-prepared clusters displayed an absorption shoulder near 400 nm and an emission peak at 622 nm upon excitation at 400 nm, closely matching those of the Au_29–43_(GSH)_27–37_ clusters (Figure S1). Matrix-assisted laser desorption/ionization time-of-flight mass spectrometry (MALDI–TOF MS) analysis of the as-prepared clusters revealed multiple peaks corresponding to the loss of gold and sulfur atoms, with *m*/*z* spacings of 197 and 32, respectively (Figure S2).^37^ These characteristic mass differences indicate the presence of both Au–S and Au–Au bonding in the as-prepared clusters. From the absorption features, emission wavelength, and MALDI-TOF MS data, it was confirmed that the as-prepared clusters correspond to the Au_29–43_(GSH)_27–37_ clusters.

We next characterized the photophysical properties of the as-synthesized Au_29–43_(GSH)_27–37_ clusters, revealing a quantum yield (QY) of 1.29% (rhodamine B as reference), an average intensity lifetime of 2.02 μs, and an amplified lifetime of 0.53 μs (Figure S3). Upon incubation of the clusters with various concentrations of Tb^3+^ in 10 mM HEPES buffer (pH 7.0), their luminescence progressively increased with Tb^3+^ concentration and reached saturation at 0.01 M Tb^3+^ (Figure 2A). At this saturation point, the clusters exhibited a 7-fold enhancement in luminescence, an increased QY of 9.14%, a prolonged average intensity lifetime of 7.33 μs, and a 16 nm blue shift in the emission maximum. These observations confirm that the AIEE of the Au_29–43_(GSH)_27–37_ clusters occurs upon the addition of Tb^3+^.^33^ The remarkable increase in QY upon Tb^3+^ saturation is attributed to aggregation-induced restriction of intramolecular motions, which suppresses nonradiative decay, and strengthens aurophilic interactions between adjacent Au^+^, both of which promote more efficient radiative emission through triplet metal-centered states. Concurrently, the hydrodynamic diameter of the Au_29–43_(GSH)_27–37_ clusters increased steadily as the Tb^3+^ concentration rose from 0.0001 to 0.001 M and remained nearly constant between 0.001 and 0.01 M Tb^3+^ (Figure 2B). After 2-day incubation to establish equilibrium between dispersion and precipitation, the supernatant from the Tb^3+^–Au_29–43_(GSH)_27–37_ mixtures was detected by UV–vis absorption spectroscopy. The absorbance of the supernatant at 400 nm dramatically reduced as the Tb^3+^ concentration varied from 640 to 900 μM Tb^3+^ (Figure 2C), consistent with the visual observation of reduced emission from the supernatant (Figure 2D). These findings suggest that Tb^3+^ acts as a bridging ion to promote intercluster association, tuning the photophysical and colloidal properties of the Au_29–43_(GSH)_27–37_ clusters. A previous study revealed that the critical salt concentration (CSC) occurs at the inflection point of the titration curve when metal ions act as the titrant for the nanoparticles.^38^ Thus, the CSC for Tb^3+^-induced precipitation of the Au_29–43_(SG)_27–37_ clusters was determined to be 640 μM Tb^3+^. It is worth emphasizing that the free energy of the dispersed nanoclusters and the aggregated nanoclusters are equal at the CSC point, meaning that the electrostatic repulsion balances van der Waals attractions and bridging interactions.

**Figure 2:**
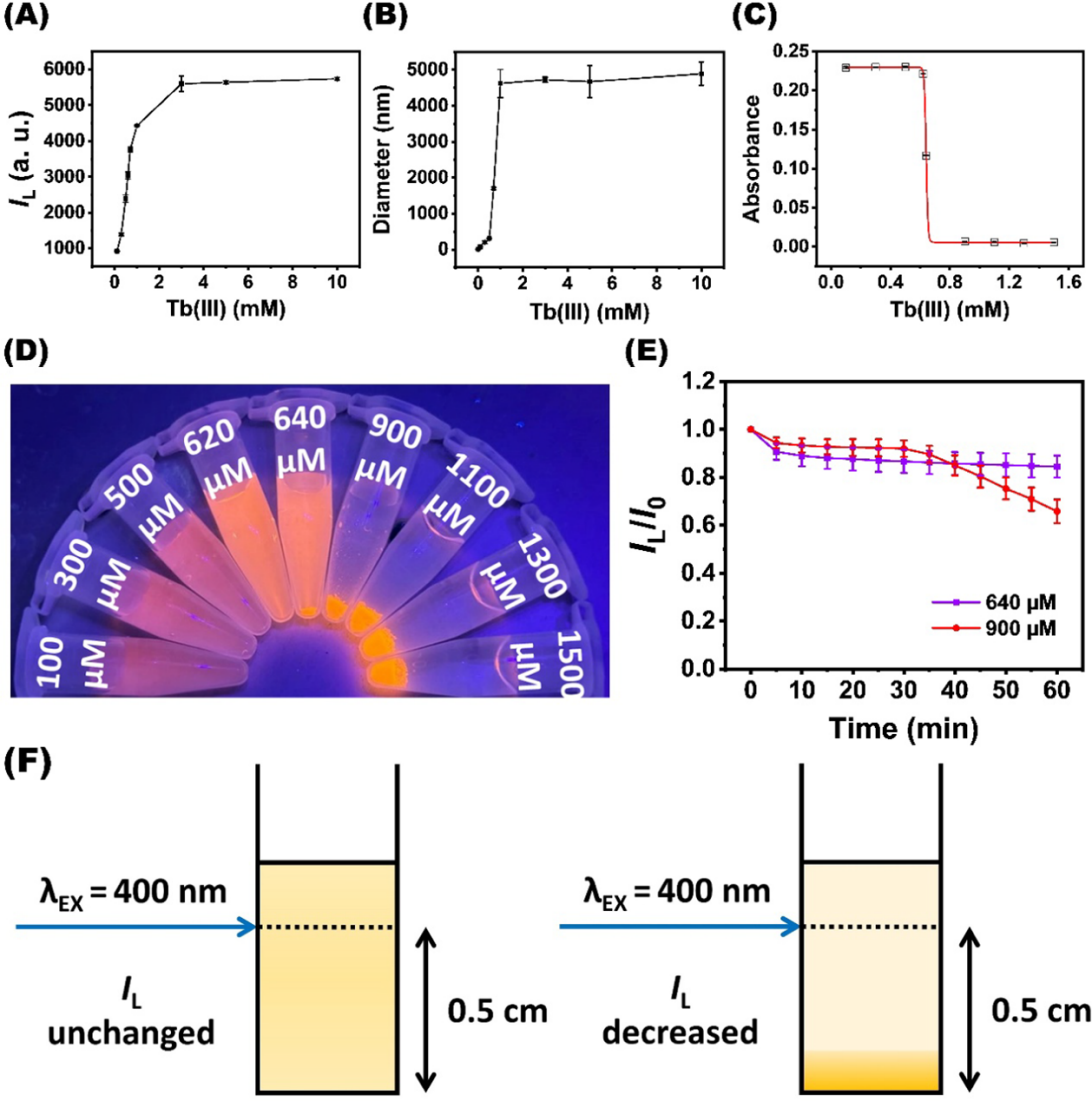
Photophysical and colloidal characterization of Tb^3+^-induced aggregation of the Au_29–43_(GSH)_27–37_ clusters in 10 mM HEPES (pH 7.0). (A) Change in the luminescence intensity of the clusters at 622 nm in the presence of increasing Tb^3+^ concentration (*I*_L_ is the luminescence intensity at a given concentration). (B) Variation in hydrodynamic diameter of the clusters as a function of Tb^3+^ concentration. (C) The absorbance of the supernatant at 400 nm obtained from incubating different Tb^3+^ concentration with the Au_29–43_(GSH)_27–37_ clusters for 2 days’ incubation. (D) Corresponding photographs of the supernatants in (C) under UV light illumination. (E) Time-dependent luminescence of the Au_29–43_(GSH)_27–37_ clusters in the presence of 640 and 900 μM Tb^3+^ under 400 nm excitation (∼0.5 cm above the cuvette bottom; the normalized luminescence intensity was expressed as *I*_L_/*I*_0_, where *I*_L_ is the luminescence intensity at a given time, and *I*_0_ is the initial luminescence intensity). (F) Schematic illustration showing the excitation beam focused ∼0.5 cm above the cuvette bottom, where precipitation reduces the aggregate density in the illuminated region.

The luminescence response of the Tb^3+^–Au_29–43_(GSH)_27–37_ mixture is inherently connected to its dispersion state in solution. When the degree of Tb^3+^-induced aggregation of the Au_29–43_(GSH)_27–37_ clusters exceeds the CSC value, the aggregates can grow large enough to sediment due to gravity. Since the excitation beam in our commercial fluorometer is focused approximately 0.5 cm above the cuvette bottom, any precipitation event reduces the number of aggregates within the illuminated region (Figure 2F). Therefore, time-dependent luminescence monitoring can be used to evaluate the dispersion stability of nanoclusters in the presence of Tb^3+^. At the CSC, the luminescence intensity of the Tb^3+^–Au_29–43_(GSH)_27–37_ mixture displays only a slight reduction under continuous excitation (Figure 2E). This result indicates that the aggregated nanoclusters at the CSC are sufficiently stable to resist sedimentation during the measurement. Additionally, the aggregated nanoclusters exhibited strong photobleaching resistance under continuous 400 nm excitation. Once the Tb^3+^ concentration was increased to 900 μM, the luminescence intensity of the Tb^3+^–Au_29–43_(GSH)_27–37_ mixture decreased sharply after 30 min of excitation. Clearly, the precipitation event removes a fraction of the aggregated nanoclusters from the excitation zone. This decline reflects that, above the CSC level, the combined effects of van der Waals attractions between nanoclusters and bridging interactions mediated by Tb^3+^ outweigh electrostatic repulsion between nanoclusters. In short, we point out the importance of optimizing Tb^3+^ concentration for fabricating the nanocluster aggregate-based sensors.

### 3.2 Linking AIEE Mechanisms with DLVO Theory

Metal cations such as Ce^3+^, Ag^+^, and Zn^2+^ can greatly boost the luminescence of GSH-AuNCs.^29,39,40^ The widely accepted mechanism is that the carboxylate groups of the capped GSH electrostatically attract and/or coordinate with metal ions, functioning as ionic bridges to assemble individual clusters into tightly packed aggregates.^41^ The aggregation stiffens the ligand shell of GSH-AuNCs, restricting intramolecular movement of GSH, inhibiting nonradiative decay channels, and ultimately increasing QY. However, the above explanation does not fully capture all aspects of AIEE, particularly in terms of its driving forces. The DLVO theoretical model is commonly used to elucidate colloidal aggregation behavior since it can interpret the interplay of forces between colloidal particles.^42^ It combines two principal interactions—van der Waals attraction and electrostatic repulsion—and thus serves as a framework for interpreting the driving forces behind AIEE.^42,43^ In the case of GSH-AuNCs, the electrostatic attraction between the metal cations and the carboxylate groups of GSH plays an equally important role in promoting aggregation. This attractive force, often referred to as a bridging interaction, should also be incorporated into the AIEE mechanism.^38^ Joo’s group developed a DLVO theory-based model to clarify the aggregation mechanism of gold nanoparticles.^44^ The model introduces the van der Waals interaction energy (*u*_vdW_) and the electrostatic interaction energy (*u*_ES_) between nanoparticles, allowing the simulation of the total interaction energy (*u*_total_) under various conditions. Based on DLVO theory, we model two GSH-Au nanoclusters as identical spheres of fixed diameter, separated by a uniform gap consisting of two GSH ligand shells and Tb^3+^ ions in both dispersed and aggregated states. Although, in principle, aggregated nanoclusters could be regarded as new particles for calculating the *u*_vdW_ value, the aggregation process in this system is primarily governed by coordination interactions (as discussed later). A similar assumption was adopted by Wang et al. in their study on the interactions between 11-mercaptoundecanoic acid-modified gold nanoparticles and monovalent cations.^38^ By fixing the particle diameter of the GSH-AuNCs, we effectively normalize the geometry of the simulated system. Thus, trends in total interaction energy reflect only changes in electrostatic repulsion, van der Waals attraction, and bridging forces, rather than size-dependent artifacts. A similar assumption has been adopted in studies investigating the interactions between citrate-capped gold nanoparticles and monovalent ions.^38,45,46^ While this approach represents an idealized simplification of the actual system, it serves as an intuitive tool for the analysis of interaction trends and stability changes.

The van der Waals interaction energy between two GSH–AuNCs can be approximated by

2 $$u_{vdW}=-\frac{A}{3}\left[\frac{{R_{c}}^{2}}{d^{2}-4{R_{c}}^{2}}+\frac{{R_{c}}^{2}}{d^{2}}+\frac{1}{2}\;\ln\left(1-\frac{4{R_{c}}^{2}}{d^{2}}\right)\right]$$

where *d* is the distance between the center of clusters, *A* is the Hamaker constant of gold in water (4 × 10^–19^ J), and *R*_c_ is the radius of Au_29–43_(GSH)_27–37_. The elemental composition and number of the Au_29–43_(GSH)_27–37_ clusters are slightly larger than those of the Au_25_ (GSH)_18_ clusters, whose metallic core size corresponds to approximately 1.0 nm.^47^ Moreover, GSH-capped gold nanoparticles with a particle size of 1.7 nm contain approximately 201 gold atoms.^47^ Thus, the core size of Au_29–43_(GSH)_27–37_ clusters is estimated to be approximately 1.2 nm, with a corresponding *R*_c_ of about 0.6 nm (Figure 3A). The center-to-center distance between two clusters is given by

3 $$d=2\times \left(R_{c}+\delta +L\right)$$

where δ is the length of the GSH ligand and *L* is the salt-bridge length. Based on Python simulations of the orientation and conformation of GSH relative to the gold core, a previous study estimated the δ distance from the sulfur atom to the most distant oxygen atom of the terminal carboxylate group in GSH to be approximately 1.05 nm.^36^ Su et al. demonstrated that the average Tb–O bond length in the crystal structure of a carboxylate complex, corresponding to the *L* value used here, is 0.245 nm.^48^ Thus, the calculated *d* value is 3.79 nm, and the resultant *u*_vdW_ value is −1.31 × 10^–23^ J. The term *x* represents the surface-to-surface distance between two clusters:

4 x=δ+L

**Figure 3:**
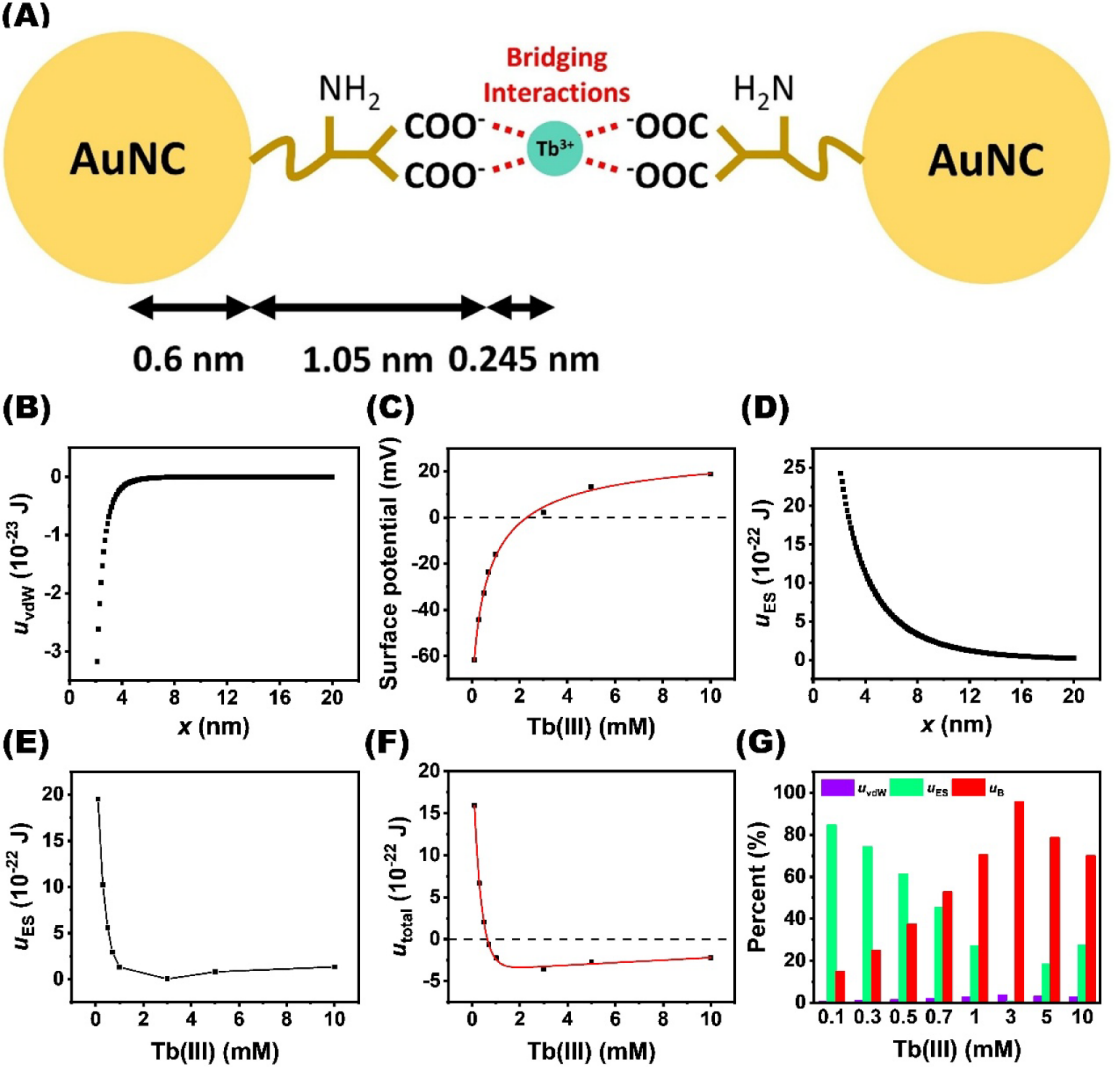
Evaluation of interparticle interaction energies governing Tb^3+^-induced aggregation of the Au_29–43_(GSH)_27–37_ clusters. (A) Schematic representation estimating the distances of the inorganic core size, capping GSH ligands, and Tb^3+^ bridging between two Au_29–43_(GSH)_27–37_ clusters. (B) Calculated van der Waals interaction energy (*u*_vdw_) between two Au_29–43_(GSH)_27–37_ clusters as a function of their separation distance. (C) Surface potential (ϕ_s_) values of the Au_29–43_(GSH)_27–37_ clusters at different Tb^3+^ concentrations. (D) Calculated electrostatic repulsion energy (*u*_ES_) between two Au_29–43_(GSH)_27–37_ clusters as a function of their separation distance. (E) Correlation between the calculated *u*_ES_ values and Tb^3+^ concentrations. (F) Total interaction energy (*u*_total_) between two Au_29–43_(GSH)_27–37_ clusters as a function of Tb^3+^ concentration. (G) Relative contributions of *u*_vdW_, *u*_ES_, and *u*_B_ at different Tb^3+^ concentrations.

As the *x* value increases, the *u*_vdW_ value gradually diminishes (Figure 3B). It is evident that the *u*_vdW_ value is highly dependent on the distance between two clusters. We next evaluated the *u*_ES_ value of two clusters in 10 mM HEPES (pH 7.0). The Debye screening length (κ^–1^) of the proposed system can be obtained according to the following equation:

5 $$\kappa^{-1}=\sqrt{\frac{\varepsilon \varepsilon_{0}k_{B}T}{2N_{A}1000Ie^{2}}}$$

where *ε* is relative permittivity (78.41), *ε*_0_ is vacuum permittivity (8.854 × 10^–12^ F/m), *k*_B_ is Boltzmann constant (1.68 × 10^–23^ J/K), *T* is absolute temperature, *N*_A_ is Avogadro constant (6.02 × 10^23^ mol^–1^), *I* is ionic strength, and *e* is elementary charge (1.6 × 10^–19^ C). Table S1 displays the calculated ionic strength and the resultant Debye screening length. Although the zeta potential can be measured directly, it only represents the attenuated potential after passing through the Stern layer and part of the diffusion layer, and thus cannot accurately reflect the surface charge of the particle. Based on the zeta potential (ζ) of the Au_29–43_(GSH)_27–37_ clusters at various Tb^3+^ concentrations and the corresponding Debye length, their surface potential (ϕ_s_) is calculated using the following Gouy–Chapman model:^49^

6 $$\phi_{s}=\frac{4k_{B}T}{ze}\;\tanh^{-1}\left(\tanh\left(\frac{ze\zeta }{4k_{B}T}\right)\times \exp\left(\kappa s\right)\right)$$

where *z* is the valence of the ion, *s* is the distance between the particle surface and the slipping plane. The *s* value is reported to be approximately 5–6 Å.^49^ Table S2 also shows the measured ζ value and the resultant ϕ_s_ value at different Tb^3+^ concentration. As indicated in Figure 3C, the negative surface charges of the Au_29–43_(GSH)_27–37_ clusters progressively decreased with increasing Tb^3+^ concentration, reaching neutrality at 2300 μM and becoming positively charged at 3000 μM. This result corroborates that Tb^3+^ can mask the surface charge of the clusters through bridging interaction and can even reverse their charge polarity at sufficiently high concentrations.^50^ Since the *κR*_c_ value of 0.0980 is smaller than 5, the following DLVO theory-related equations are selected to determine the *u*_ES_ value.^44^

7 $$Y=\frac{8\;\tanh\left(\frac{e\phi_{s}}{4k_{B}T}\right)}{1+{\left[1-\frac{2\kappa R_{c}+1}{{\left(\kappa R_{c}+1\right)}^{2}}\;\tanh^{2}\left(\frac{e\phi_{s}}{4k_{B}T}\right)\right]}^{1/2}}$$

8 $$u_{ES}=4\pi \varepsilon \varepsilon_{0}{R_{c}}^{2}Y^{2}{\left(\frac{k_{B}T}{e}\right)}^{2}\frac{\exp\left(-\kappa x\right)}{x+2R_{c}}$$

where *Y* is a correction factor related to the surface potential, which is used to describe the electrostatic double-layer interaction energy between particles. Figure 3D shows that an increase in the *x* value leads to a gradual reduction in the *u*_ES_ value. Because the electrostatic repulsion is influenced by the ion-screening effect, its magnitude decreases as the interparticle distance increases. The calculated *u*_ES_ values of two clusters at different Tb^3+^ concentrations are listed in Table S2. Plotting the *u*_ES_ value as a function of Tb^3+^ concentration reveals a trend consistent with that of the zeta potential, as shown in Figure 3E. Under the condition of a fixed particle size, the *u*_ES_ value of two clusters drops continuously with increasing Tb^3+^ concentration and reaches its minimum at 3000 μM Tb^3+^. This finding signifies that the addition of Tb^3+^ effectively weakens the electrostatic repulsion between the clusters through bridging interactions. A slight increase in the *u*_ES_ value between two clusters is observed above 3000 μM Tb^3+^. Evidently, the electrostatic repulsion between two clusters is re-established due to the excessive Tb^3+^ adsorption. If the system strictly follows the DLVO model, the total interaction energy can be expressed as

9 $$u_{\mathrm{total}}=u_{\mathrm{vdW}}+u_{\mathrm{ES}}$$

If the GSH–AuNCs are on the verge of precipitation, the *u*_total_ value between two clusters will approach zero due to the balance between the *u*_vdw_ and *u*_ES_ values. After plotting the *u*_total_ value against the Tb^3+^ concentration and fitting the data with a double-exponential function, the intersection point of the fitted curve with the *y* = 0 axis corresponds to a theoretical CSC of approximately 2000 μM (Figure S4A). However, this theoretical CSC value does not closely match the experimentally determined CSC of 640 μM. Furthermore, Figure S4B displays the relative contributions of *u*_vdW_ and *u*_ES_ to the *u*_total_ value at different Tb^3+^ concentrations. The *u*_total_ value is dominated by electrostatic repulsion between two clusters, whereas van der Waals attraction exceeds electrostatic repulsion only at a surface potential approaching zero (i.e., 3000 μM Tb^3+^). Nevertheless, the cluster precipitation begins to occur at 640 μM. This discrepancy suggests that the classical DLVO model is insufficient to fully describe the aggregation behavior of the Au_29–43_(GSH)_27–37_ clusters. This result implies that the bridging interaction should be incorporated into the aggregation behavior of the Au_29–43_(GSH)_27–37_ clusters. Wang et al. modified the DLVO theory by introducing the bridging interaction energy (*u*_B_).^38^ This additional interaction allows our model to correctly reflect the effect of Tb^3+^ on the interparticle interactions between the Au_29–43_(GSH)_27–37_ clusters. Thus, the DLVO model can be modified using the following equation:

10 $$u_{\mathrm{total}}=u_{\mathrm{vdW}}+u_{ES}+u_{B}$$

Since the *u*_B_ value is highly linked to dissociation energy (Δ*G*_d_), it is assumed to be linearly proportional to its negative value, expressed as

11 $$u_{B}=-f\Delta G_{d}$$

where *f* serves as a calibration constant to adjust the calculated bridging interaction energy. Notably, this equation represents the simplest approximation, without accounting for the influences of electronic effects, steric hindrance, or bond rearrangements on the cluster geometry.^38^ It is well-known that Δ*G*_d_ can be expressed thermodynamically as

12 $$\Delta G_{d}=-RT\;\ln\;K_{d}$$

where *R* is the gas constant and *T* is the absolute temperature. Therefore, the determination of *K*_d_ is a crucial step in quantifying *u*_B_. The *K*_d_ value between the Au_29–43_(GSH)_27–37_ clusters and Tb^3+^ is calculated to be 6.41 × 10^–4^ M (Figure S5), meaning that the Δ*G*_d_ value is 3.03 × 10^–20^ J/atom. When the proportionality constant *f* is adjusted to 0.0113, the intersection of the fitted *u*_total_ at *y* = 0 axis is found to be 640 μM (Figure 3F). The obtained value coincides with the experimentally determined CSC. Under this condition, the *u*_B_ value is determined to be −3.42 × 10^–22^ J. It is clear that inclusion of the bridging interaction term (*u*_B_) reconciles the theoretical prediction with the observed onset of the cluster aggregation. Overall, Table S3 and Figure 3G reveal the values of three types of interactions and the relative contributions of those to the *u*_total_ values at various Tb^3+^ concentrations. At low Tb^3+^ concentrations, strong electrostatic repulsion keeps the clusters dispersed. An increase in the Tb^3+^ concentration progressively reduces the contribution of electrostatic repulsion to the total interaction energy, while the proportion of bridging interactions gradually rises. At 640 μM Tb^3+^, the sum of van der Waals and bridging interactions is equal to the electrostatic repulsion, driving the onset of precipitation. Beyond this concentration, adding more Tb^3+^ makes bridging interactions the main driving force, causing extensive aggregation and forming larger clusters.

### 3.3 Ratiometric Sensing of Lf

Previous studies have demonstrated the specific interaction between Tb^3+^ and Lf,^51–53^ prompting us to examine the effect of Lf on the luminescence of the Tb^3+^–Au_29–43_(GSH)_27–37_ aggregates. Prior to evaluating this hypothesis, we first investigated whether Lf is capable of forming a complex with Tb^3+^. Figure S6A reveals that the emission peak of Tb^3+^ in the Lf-Tb^3+^ complexes at 554 nm incrementally intensified with increasing Lf concentration. Plotting the intensity of the emission peak versus the Lf concentration generates a linear calibration curve (*R* ^2^ = 0.9981) for quantifying 0.5–3.0 mg/mL Lf with a limit of detection (LOD; signal-to-noise ratio of 3) corresponding to 150 μg/mL (Figure S6B). Additionally, the average relative standard deviation (RSD) of the measured signal is determined to be 6.2%. The Hill equation analysis yielded a binding constant of 4.56 × 10^4^ M^–1^ for the Tb^3+^–Lf complex (Figure S6C). These observations corroborate strong binding of Tb^3+^ to Lf, which is in agreement with spectroscopic evidence obtained from previous studies.^51–53^ The subsequent study focused on the detection of Lf using the Tb^3+^–Au_29–43_(GSH)_27–37_ aggregates in 10 mM HEPES (pH 7.0). The introduction of 3.0 mg/mL Lf to a solution of the luminescent aggregates (*C*_Au_ = 0.087 mg/mL) caused a reduction in their luminescence intensity and lifetime (Figure 4A and B). Under identical conditions, the hydrodynamic diameter of the luminescent aggregates decreased from 2273 to 178 nm (Figure 4C), while their zeta potential shifted from 3.9 to −9.4 mV (Figure 4D). The transmission electron microscopy (TEM) images reveal an increasing particle size trend in the following order: Au_29–43_(GSH)_27–37_ clusters < Lf-cluster aggregates mixture < Tb^3+^–Au_29–43_(GSH)_27–37_ aggregates (Figure 4E–G). Taken together, these findings signify that Lf triggers the liberation of Tb^3+^ from the luminescent aggregates, converting them from the aggregated state to the dispersed state, as illustrated in Figure S7. We next evaluated the sensitivity of the luminescent aggregates toward Lf. As the Lf concentration changed from 0 to 3.0 mg/mL, the luminescence of the Tb^3+^–Au_29–43_(GSH)_27–37_ aggregates steadily diminished (Figure 5A). The luminescent aggregates yield a linear calibration curve (*R* ^2^ = 0.9962) when the luminescence intensity at 607 nm is plotted against the Lf concentration over the range of 0.01–3.0 mg/mL (Figure 5B). Using the luminescent aggregates, the LOD of Lf is estimated to be 8.5 μg/mL, with an average relative standard deviation of 3.6% for the measured signal. Although the Tb^3+^–Au_29–43_(GSH)_27–37_ aggregates exhibit a strong luminescence turn-off response toward Lf, turn-off systems are inherently less observable and are more susceptible to interference in complex matrices.^54^ Additionally, single-wavelength sensing is susceptible to errors from light source instability, probe concentration variations, and matrix effects.^55^

**Figure 4:**
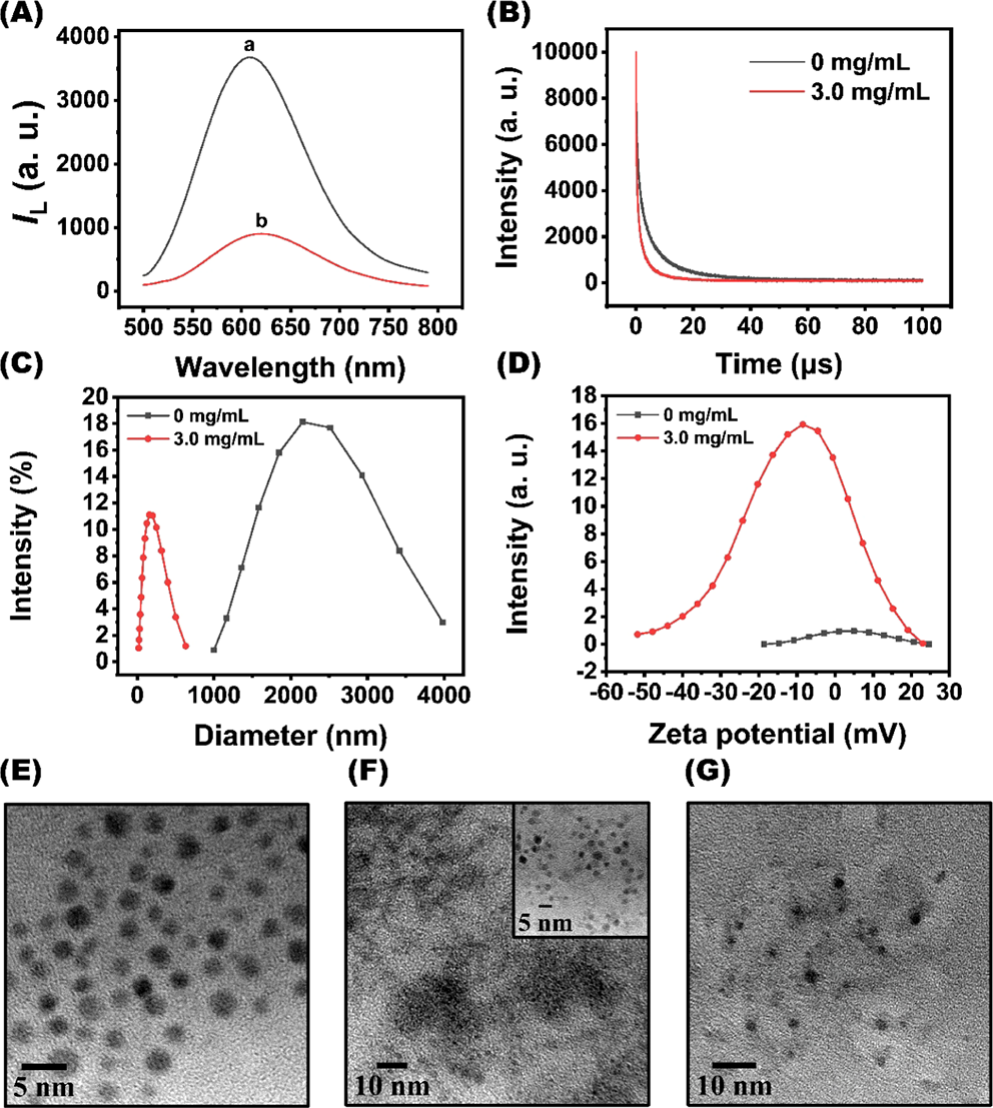
Lf-mediated disassembly of Tb^3+^–Au_29–43_(GSH)_27–37_ aggregates. (A) Luminescence spectra of (a) Tb^3+^–Au_29–43_(GSH)_27–37_ aggregates, and (b) Lf-aggregate mixture in the presence of 3.0 mg/mL Lf. (B) Time-resolved luminescence decay profiles of the aggregates (*C*_Au_ = 0.087 mg/mL) as a function of the Lf concentration. (C) Hydrodynamic diameter and (D) zeta potential of the aggregates in the absence and presence of 3.0 mg/mL Lf. (E–G) TEM images of the Au_29–43_(GSH)_27–37_ clusters, the Tb^3+^–Au_29–43_(GSH)_27–37_ aggregates, and the Lf-aggregate mixture. (A–G) The Tb^3+^–Au_29–43_(GSH)_27–37_ aggregates were incubated with Lf in 10 mM HEPES (pH 7.0) for 10 min.

**Figure 5:**
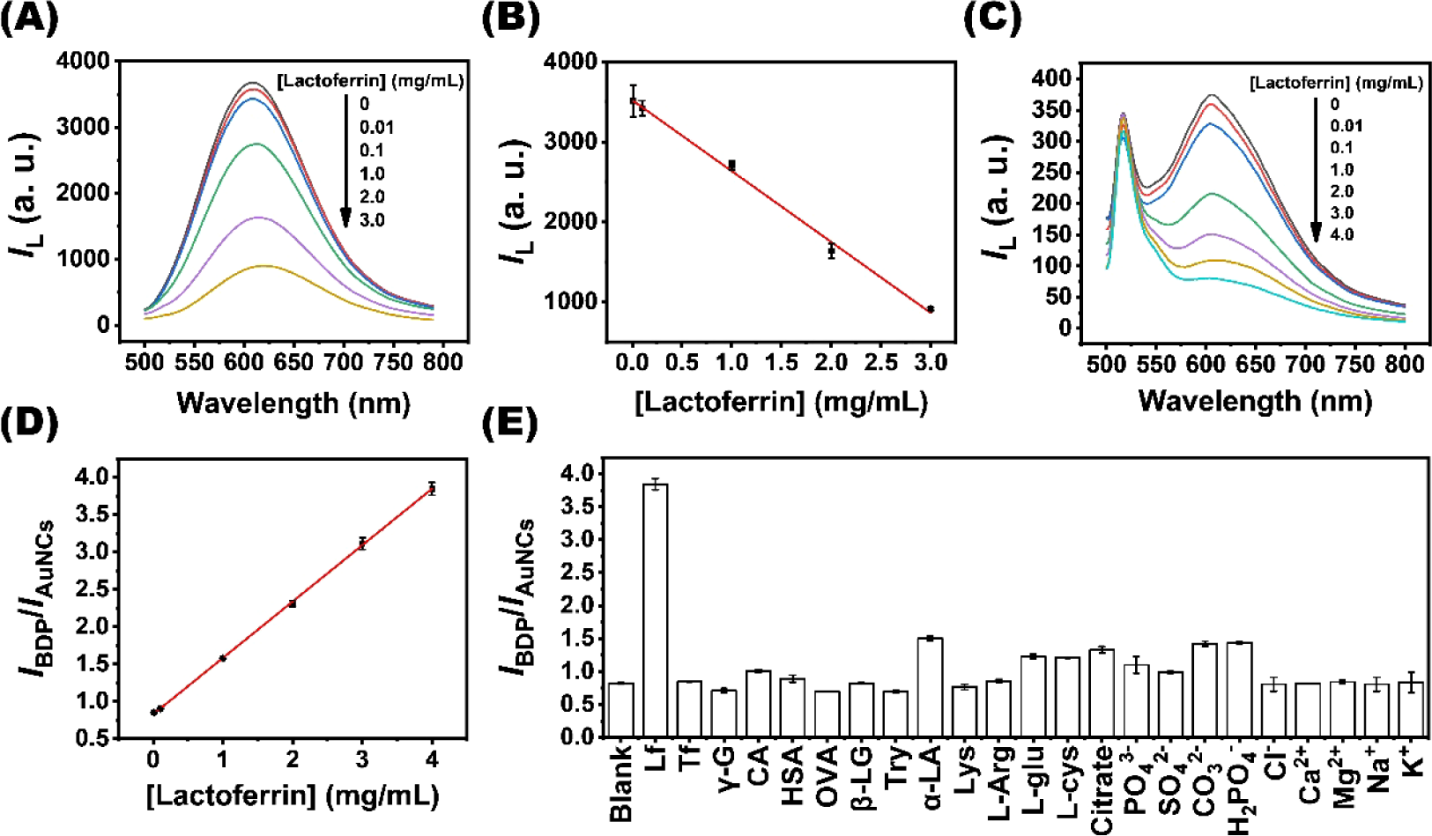
Sensing of Lf with the Tb^3+^–Au_29–43_(GSH)_27–37_ aggregates and BDP-FL-conjugated AIEE dots. (A) Luminescence spectra of the Tb^3+^–Au_29–43_(GSH)_27–37_ aggregates in the presence of increasing Lf concentrations (0–3.0 mg/mL). (B) Corresponding calibration curve of luminescence intensity at 607 nm versus Lf concentration. (C) Luminescence spectra of the BDP-FL-conjugated AIEE dots in the presence of increasing Lf concentrations (0–4.0 mg/mL). (D) Corresponding calibration curve of the *I*_BDP_/*I*_AuNCs_ ratio against the Lf concentration. (E) The *I*_BDP_/*I*_AuNCs_ ratio obtained from incubating the BDP-FL-conjugated AIEE dots with proteins, amino acids, anions, and metal ions. Tf, γ-G, CA, HSA, OVA, β-LG, Try, α-LA, Lys, l-Arg, l-glu, and l-cys correspond to transferrin, γ-globulins, conalbumin, human serum albumin, ovalbumin, β-lactoglobulin, trypsin, α-lactalbumin, lysozyme, l-arginine, l-glutamine, and l-cysteine, in sequence. The concentration of each analyte is 50 μM.

In response to this obstacle, we performed the conjugation of BDPP-FL-N-hydroxysuccinimide ester (NHS) to the amine groups of the Au_29–43_(GSH)_27–37_ clusters via 1-ethyl-3-(3-(dimethylamino)­propyl)­carbodiimide hydrochloride (EDC) and NHS coupling chemistry. Unlike fluorescein isothiocyanate, the fluorescence intensity of BDP-FL is insensitive to changes in solution pH. The conjugated BDP-FL in the Au_29–43_(GSH)_27–37_ clusters can serve as the reference channel in ratiometric sensing of Lf. The resultant products were purified through the removal of excess BDP-FL with a centrifugal filtration column. The fluorescence of the collected filtrate faded to negligible levels after repeated washing and centrifugation steps (Figure S8). The retentate containing the purified BDP-FL-conjugated clusters was analyzed by gel permeation chromatography (Figure S9) and compared with the unpurified clusters. The unpurified clusters displayed two peaks in the chromatogram, whereas the purified clusters showed only a single peak. Since the retention time of purified clusters resembles that of the Au_29–43_(GSH)_27–37_ clusters, we confirm the successful conjugation of BDP-FL to the Au_29–43_(GSH)_27–37_ clusters. As indicated in Figure S10, the obtained BDP-FL-conjugated clusters have a hydrodynamic diameter of 3.3 ± 1.1 nm, a zeta potential of −18.5 mV, dual-emission peaks of 517 nm (*I*_BDP_) and 630 nm (*I*_AuNCs_) upon excitation at 450 nm. The selection of this excitation wavelength is based on maximizing both the fluorescence signal of BDP-FL and the luminescence signal of the Au_29–43_(GSH)_27–37_ clusters (Figure S11A). The QY of Au_29–43_(GSH)_27–37_ clusters in the BDP-FL-conjugated clusters is 1.86%, and their average and amplified intensity lifetimes separately correspond to 2.12 and 0.54 μs (Figure S11B), which are consistent with those of the unconjugated Au_29–43_(GSH)_27–37_ clusters. This observation reflects that minimal fluorescence resonance energy transfer occurs from BDP-FL molecules to the Au_29–43_(GSH)_27–37_ clusters. Under 450 nm continuous irradiation, the fluorescence intensity of BDP remained stable, and the luminescence intensity of the AuNCs reached a steady level after 5 min (Figure S11C). The *I*_BDP_/*I*_AuNCs_ ratio remained almost constant over the pH range of 4.0–10 in 10 mM HEPES buffer (Figure S11D). Thus, the ratiometric signal of the BDP-FL-conjugated clusters is photostable and insensitive to pH variations.

Upon addition of 3000 μM Tb^3+^, the formed Tb^3+^–BDP-FL-conjugated cluster aggregates (named BDP-FL-conjugated AIEE dots; *C*_Au_ = 0.49 mg/mL) exhibit stable luminescence intensity for 1 h without noticeable variation during the measurement (Figure S12). Accordingly, they are suitable for Lf sensing for at least 1 h without precipitation. In order to maintain comparable peak intensities between BDP-FL and the AIEE dots, the excitation wavelength was adjusted to 488 nm. Under these conditions, the aggregates possess an increased hydrodynamic diameter of 2444.7 ± 26.7 nm (Figure S13A) and a reduced zeta potential of −4.18 mV (Figure S13B). The luminescence intensity and lifetime of the Au_29–43_(GSH)_27–37_ clusters in the BDP-FL-conjugated AIEE dots enhanced progressively with increasing Tb^3+^ concentration (Figure S13C,D). At a luminescence saturation of 0.01 M Tb^3+^, the formed BDP-FL-conjugated AIEE dots exhibited an approximately 7-fold enhancement in intensity and an improved QY of 9.71%, compared to that of the BDP-FL-conjugated clusters. Time-resolved fluorescence spectroscopy was employed to examine the luminescence dynamics of the BDP-FL-conjugated AIEE dots in the absence and presence of 0.01 M Tb^3+^ (Figure S14). Evidently, the luminescence lifetime of the clusters is much longer than that of BDP-FL molecules. The addition of Tb^3+^ further prolonged the lifetime of the clusters while the fluorescence lifetime of the BDP-FL remained almost unchanged. These findings indicate that Tb^3+^ mainly influences the photophysical properties of the clusters rather than BDP-FL molecules. In other words, BDP-FL conjugation rarely interferes with the Tb^3+^ -induced AIEE of the Au_29–43_(GSH)_27–37_ clusters. Upon addition of 3.0 mg/mL Lf to the BDP-FL-conjugated AIEE dots, the resulting mixture displayed a reduced hydrodynamic diameter of 170.6 ± 24.1 nm (Figure S15A) and an increased zeta potential of −15.4 mV (Figure S15B). As the Lf concentration varied from 0 to 4.0 mg/mL, we observed a consistent fluorescence intensity of BDP-FL molecules, while the luminescence intensity of the Au_29–43_(GSH)_27–37_ clusters decreased (Figure 5C). Meanwhile, the luminescence lifetime of the Au_29–43_(GSH)_27–37_ clusters in the BDP-FL-conjugated AIEE dots shortened progressively with increasing Lf concentration (Figure S16C). A linear relationship (*R* ^2^ = 0.9998) between the *I*_BDP_/*I*_AuNCs_ value and Lf concentration was obtained over the range of 0.01–4.0 mg/mL (Figure 5D). The LOD of Lf, detected by the BDP-FL-conjugated AIEE dots, is estimated to be 3.4 μg/mL, which is 2 orders of magnitude lower than that of the Lf–Tb^3+^ complexes and comparable to those of previously reported sensors (Table S4). More importantly, the average RSD of the measured ratiometric signal was lower than 1.2%, demonstrating that BDP-FL indeed serves as an internal reference to improve measurement accuracy. The selectivity of the BDP-FL-conjugated AIEE dots was assessed by substituting Lf with the same concentration of various proteins, amino acids, anions, and metal ions, one at a time. To further assess their selectivity under real-world conditions, we formulated a simulated human tear matrix containing lysozyme, human serum albumin, Ca^2+^, Mg^2+^, Na^+^, K^+^, glucose, and other major tear components at physiological concentrations.^56–60^ Considering that lactoferrin is the transferrin-family protein in human tears, secreted by the lacrimal gland at concentrations around 1.3–2.5 mg mL^–1^,^61^ we also include transferrin in our selectivity tests. As shown in Figures 5E and S16, only Lf produced a remarkable increase in the *I*_BDP_/*I*_AuNCs_ value, demonstrating that the BDP-FL-conjugated AIEE dots provide excellent selectivity toward Lf owing to the specific binding of Lf to Tb^3+^.^51^

To evaluate their practical applicability, the BDP-FL-conjugated AIEE dots were employed for Lf determination in tear samples, and the results were compared with those obtained by capillary electrophoresis. After spiking tear samples with varying concentrations of standard Lf (0.05–4.0 mg/mL) and introducing them into a solution of the BDP-FL-conjugated AIEE dots, the resulting *I*_BDP_/*I*_AuNCs_ ratio increased linearly (*R* ^2^ = 0.9995) in a concentration-dependent manner (Figure S17). The average RSD value of the measured *I*_BDP_/*I*_AuNCs_ values is lower than 2%. The difference in the slope of the calibration curve between the standard and spiked Lf is only 1.62%, and the recovery of the spiked Lf ranges from 99.98 to 101.8% (Table S5), suggesting that the proposed probe is free from the matrix effect of tear samples. In other words, the external calibration curve is suitable for determining Lf concentrations in tear samples, yielding a value of 1.4 2 ± 0.04 mg/mL. It is noted that the level of Lf falls within the normal physiological range.^56^ The concentration of Lf in tear samples, as determined by capillary electrophoresis, was 1.41 ± 0.03 mg/mL (Figure S18). In comparison with the results obtained using the proposed probe, no statistically significant differences were observed based on *t*-test and *F*-test analyses.

## 4 Conclusions

We have developed a ratiometric AIEE probe for the quantitative determination of Lf in human tears, based on the interplay among Tb^3+^ ions, Au_29–43_(GSH)_27–37_, and Lf. The BDP-FL-conjugated AIEE dots exhibited ratiometric luminescence characteristics with dual emission at 517 and 606 nm, providing a self-calibration function that minimizes matrix effects and improves the accuracy and reliability of Lf measurement. In addition, this work elucidates the mechanism of Tb^3+^-induced AIEE of the Au_29–43_(GSH)_27–37_ clusters. Since van der Waals forces are relatively weak and insufficient to trigger aggregation on their own, and the increase in Tb^3+^ concentration, while effectively reducing electrostatic repulsion between clusters, is still inadequate to induce precipitation, these findings highlight the crucial role of the salt-bridging effect in the aggregation process. Bridging interactions not only greatly exceed van der Waals forces but also overcome electrostatic repulsion at high Tb^3+^ concentrations, becoming the primary driving force for cluster aggregation and precipitation. While this study mainly focuses on the influence of lanthanide ions on nanoparticle interactions, future work will extend to other metal ions, including alkali metals (Na^+^ and K^+^), alkaline earth metals (Mg^2+^ and Ca^2+^), and transition metals (Zn^2+^ and Cd^2+^). Such a systematic exploration could clarify how differences in the structural stability and coordination ability of metal ions affect the balance among van der Waals forces, electrostatic repulsion, and bridging interactions between nanoparticles. By constructing a comprehensive metal ion interaction map, our goal is to gain deeper insights into the physicochemical basis of the AIEE mechanism, thereby developing nanomaterials with ion selectivity, tunable luminescence properties, and controllable self-assembly.

## Supplementary Material



## Notes

Experimental details, instrumental parameters, and supplementary results, including absorption and luminescence spectra of Au_29–43_(GSH)_27–37_ clusters (Figure S1), MALDI–TOF MS spectrum (Figure S2), luminescence decay and quantum yield (Figure S3), interaction energies at varying Tb^3+^ concentrations (Figure S4), and binding isotherm analysis (Figure S5); luminescence spectra of Tb^3+^–Lf complexes with calibration curves (Figure S6), schematic illustration of Lf-mediated disassembly (Figure S7), and purification of BDP-FL–conjugated clusters (Figure S8); gel permeation chromatograms (Figure S9), hydrodynamic diameter, zeta potential, and optical data (Figures S10–S13), time-resolved luminescence spectra (Figure S14), Lf-induced changes in size, charge, and lifetimes (Figure S15), and selectivity of the BDP-FL–conjugated AIEE dots in the presence of mimicking tear matrix components (Figure S16); additional luminescence spectra and calibration plots for tear sample analysis are shown (Figures S17, S18); structure of BDP-FL NHS ester (Figure S19); ionic strength and Debye length (Table S1), zeta potential, surface potential, and electrostatic energy (Table S2), the relative contributions of three types of interaction energy (Table S3), analytical performance comparisons (Table S4), and recovery with precision in tear samples (Table S5) (PDF)
