# Supplementary material for: Exploring Tb3+-Mediated Interactions with Glutathione-Capped Gold Nanoclusters to Develop a Fluorophore-Modified Ratiometric Probe toward Lactoferrin
Source: J Phys Chem B. 2025 Oct 30;129(45):11851–61. doi: 10.1021/acs.jpcb.5c06319 (PMC12621237; doi:10.1021/acs.jpcb.5c06319)
Supplement: Supplementary file 1 [file jp5c06319_si_001.pdf]

Supporting Information

Exploring Tb^{3+}-Mediated Interactions with Glutathione-Capped Gold Nanoclusters to Develop a Fluorophore-Modified Ratiometric Probe toward Lactoferrin

Chun-Hsin Kuo,[a] Bo-Yu Liu,[a] Shin-Yi Feng,[b] Cheng-Kang Chiang,[b] Ming-Mu Hsieh[c] and Wei-Lung Tseng [a, d, e*]

[a] Department of Chemistry, National Sun Yat-sen University, No. 70 Lienhai Rd., Kaohsiung 80424, Taiwan.

[b] Department of Chemistry, National Dong Hwa University, Shoufeng, Hualien 974301, Taiwan.

[c] Department of Chemistry, National Kaohsiung Normal University, No.62, Shenjhong Rd., Yanchao District, Kaohsiung City 82446, Taiwan.

[d] Center for Nanoscience & Nanotechnology, National Sun Yat-sen University, No. 70 Lienhai Rd., Kaohsiung 80424, Taiwan.

[e] School of Pharmacy, College of Pharmacy, Kaohsiung Medical University, No.100, Shiquan 1st Rd., 80708, Kaohsiung, Taiwan.

Correspondence: Dr. Wei-Lung Tseng, E-mail: tsengwl@mail.nsysu.edu.tw Fax: 011-886-7-5254644

Experimental

Chemicals. GSH (98%), HEPES (98%), sodium citrate (≥99.0%), human serum albumin (≥95%), sodium dihydrogen phosphate (98%), L-cysteine (≥98%), L-arginine (98.5-101.0%), α-lactalbumin (≥95%), human lactoferrin (90%), human transferrin (≥98%), β-lactoglobulin (≥90%), and γ-globulins (≥99%) were purchased from Sigma-Aldrich (St. Louis, MO, USA). Hydrogen tetrachloroaurate trihydrate (99.99%) was obtained from Alfa Aesar (Ward Hill, MA, USA). Terbium(III) nitrate hexahydrate (99.99+%), L-glutamine (≥99%), potassium phosphate (97%), and glucose (≥95%) were purchased from Acros Organics (Geel, Belgium). Sodium chloride (99.5%), potassium chloride (99%), and potassium carbonate (99.5%) were purchased from Showa (Tokyo, Japan). Sodium sulfate (≥99%) was obtained from Shimakyu's Pure Chemicals (Osaka, Japan). Magnesium chloride hexahydrate (99.0-102.0%) was purchased from J.T. Baker (PA, USA). Calcium chloride dehydrate (≥99%) was obtained from Aencore (Box Hill, VIC, Australia). Lysozyme (≥95%), trypsin (≥95%), ovalbumin (>80%), and conalbumin (≥90%) was purchased from MP Biomedicals (Solon, OH, USA). Boron-dipyrromethene N-hydroxysuccinimide ester (BDP–FL NHS ester, >95%) was purchased from Lumiprobe (Westminster, Maryland, USA), and its chemical structure is shown in **Figure S19**. Milli-Q ultrapure water with a resistivity of 18.2 MΩ·cm (Millipore, Hamburg, Germany) was used throughout all experiments.

Instruments. UV–Vis absorption spectra were recorded using a double-beam spectrophotometer (Cintra 10e; GBC, Victoria, Australia), and fluorescence spectra were measured with a fluorescence spectrophotometer (F-7000; Hitachi, Tokyo, Japan). Zeta potential measurements were performed using a Delsa™ Nano zeta potential and submicron particle size analyzer (Beckman Coulter Inc., USA). DLS measurements were carried out on an N5 submicron particle size analyzer

(Beckman Coulter Inc., USA), and Litesizer DLS 501 dynamic light scattering instrument (Anton Paar, Austria). TEM images were obtained using a JEM-2100 transmission electron microscope (JEOL, Japan) operated at an accelerating voltage of 200 kV. Lattice spacings were determined by performing Fourier transform analysis of the TEM images and Digital Micrograph software. The luminescence lifetime was recorded on a time-correlated single-photon counting (TCSPC) system (TimeHarp 200, PicoQuant GmbH, Berlin, Germany) equipped with a 390 nm pulsed diode laser (pulse width of tens of picoseconds). Time-resolved luminescence spectra were measured using an LP980 (Edinburgh Instruments, UK). A laboratory-built GPC system was used to verify the formation of BDP-FL–conjugated clusters. The GPC setup includes an LC pump, a UV detector, an OHpak SB-804 HQ column, and a mobile phase. The mobile phase is 150 mM phosphate buffer (pH 7.0). Capillary electrophoresis was employed to determine Lf in human tear. According to the previously reported method,[1] the total and effective lengths of a capillary were set to 61.2 and 51.2 cm, respectively. Detection (220 nm) was carried out with an ECD2600 CE UV–Vis detector (ECOM, Czech Republic), while the separation was performed at +25 kV in 50 mM phosphate buffer (pH 4.0) containing 6 M urea.

Reference

1. Chen, H.; Wang, Z.; Fan, F.; Shi, P.; Xu, X.; Du, M.; Wang, C., Analysis Method of Lactoferrin Based on Uncoated Capillary Electrophoresis. *eFood* **2021,** *2* (3), 147-153.

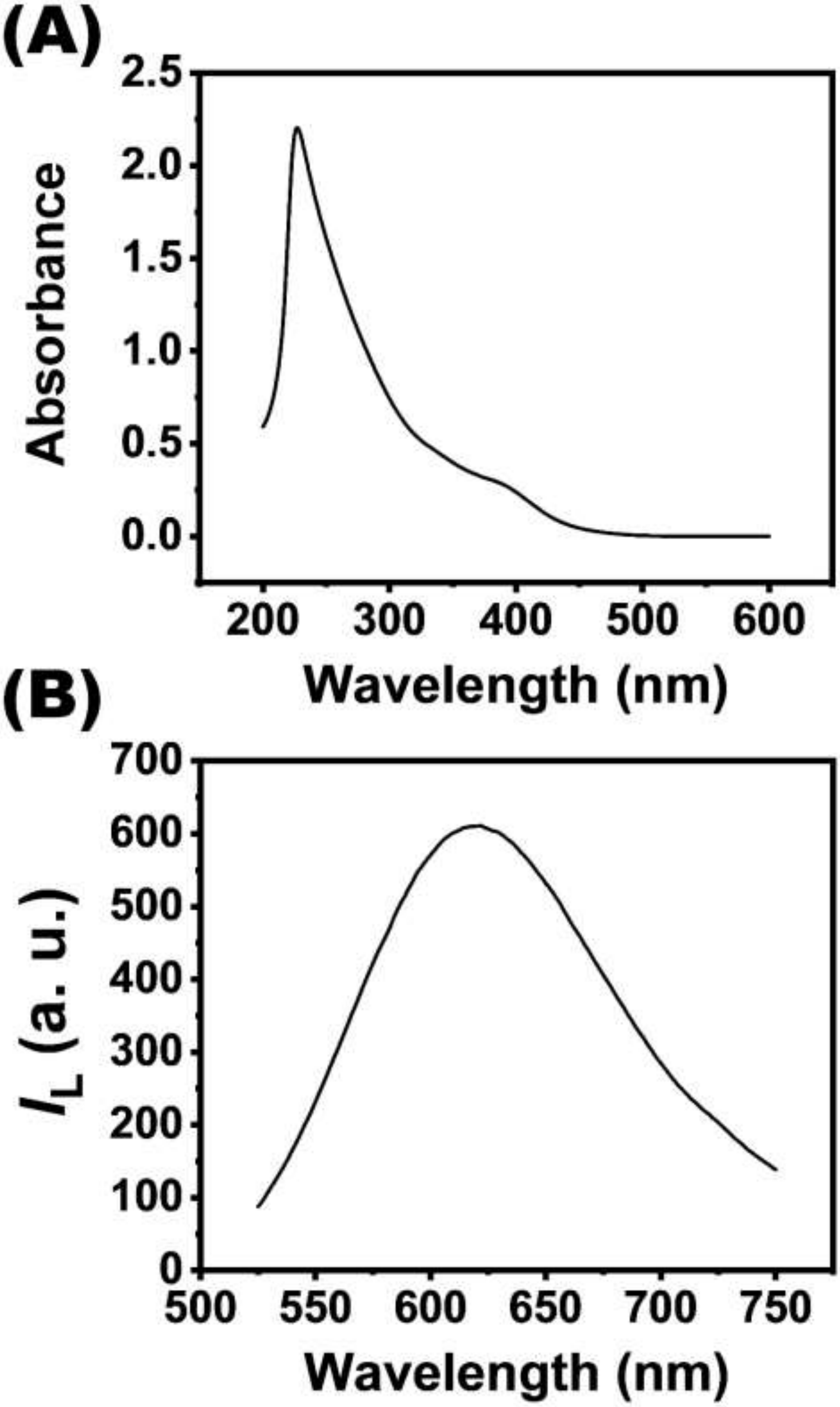

Figure S1. (A) Absorption and (B) luminescence spectra of the as-prepared $Au_{29-43}(GSH)_{27-37}$ clusters in 10 mM HEPES (pH 7.0).

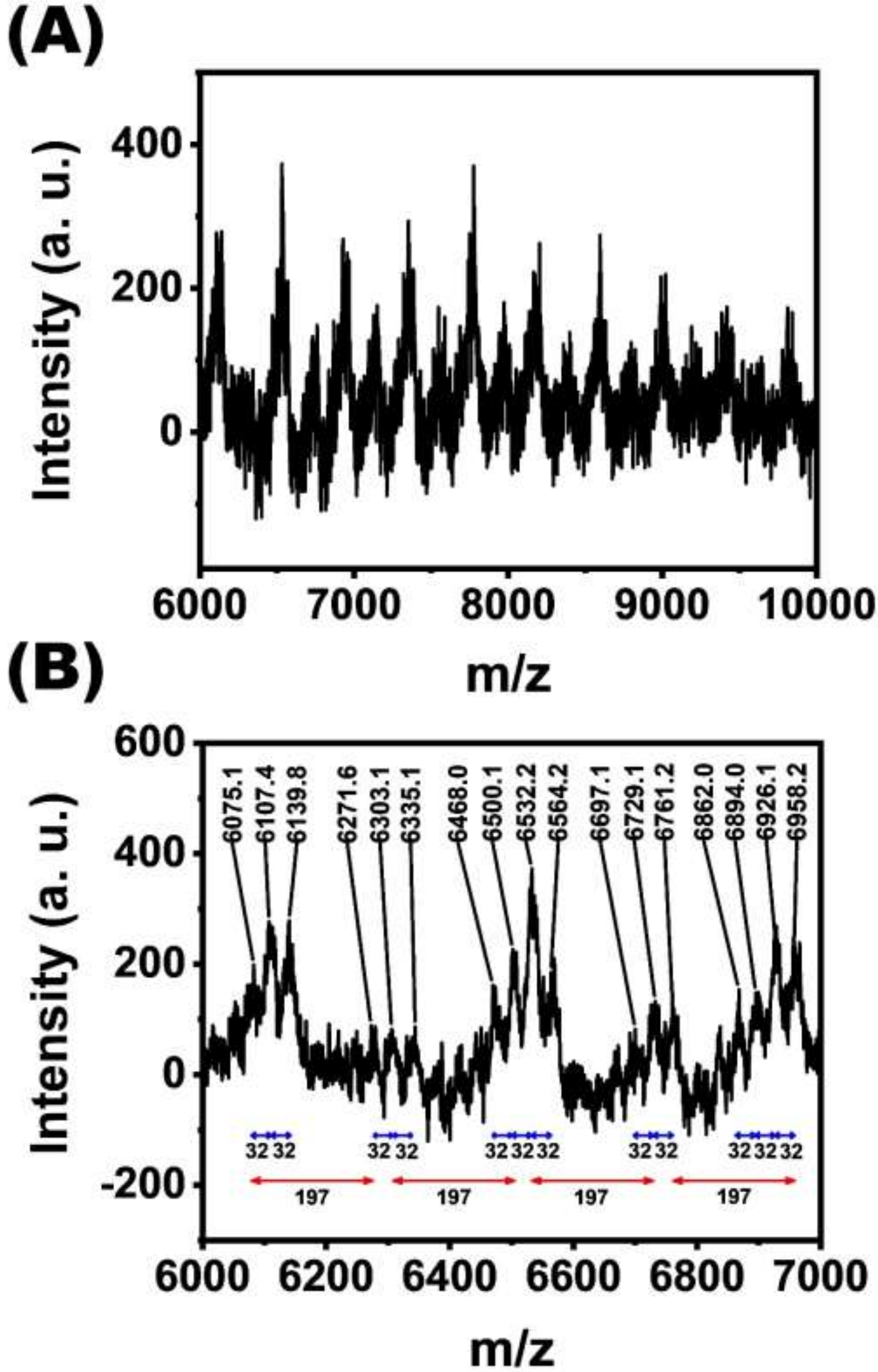

Figure S2. MALDI–TOF MS spectrum of the as-prepared $Au_{29-43}(GSH)_{27-37}$ clusters, with the ranges of (A) 6000–10000 m/z and (B) 6000–7000 m/z.

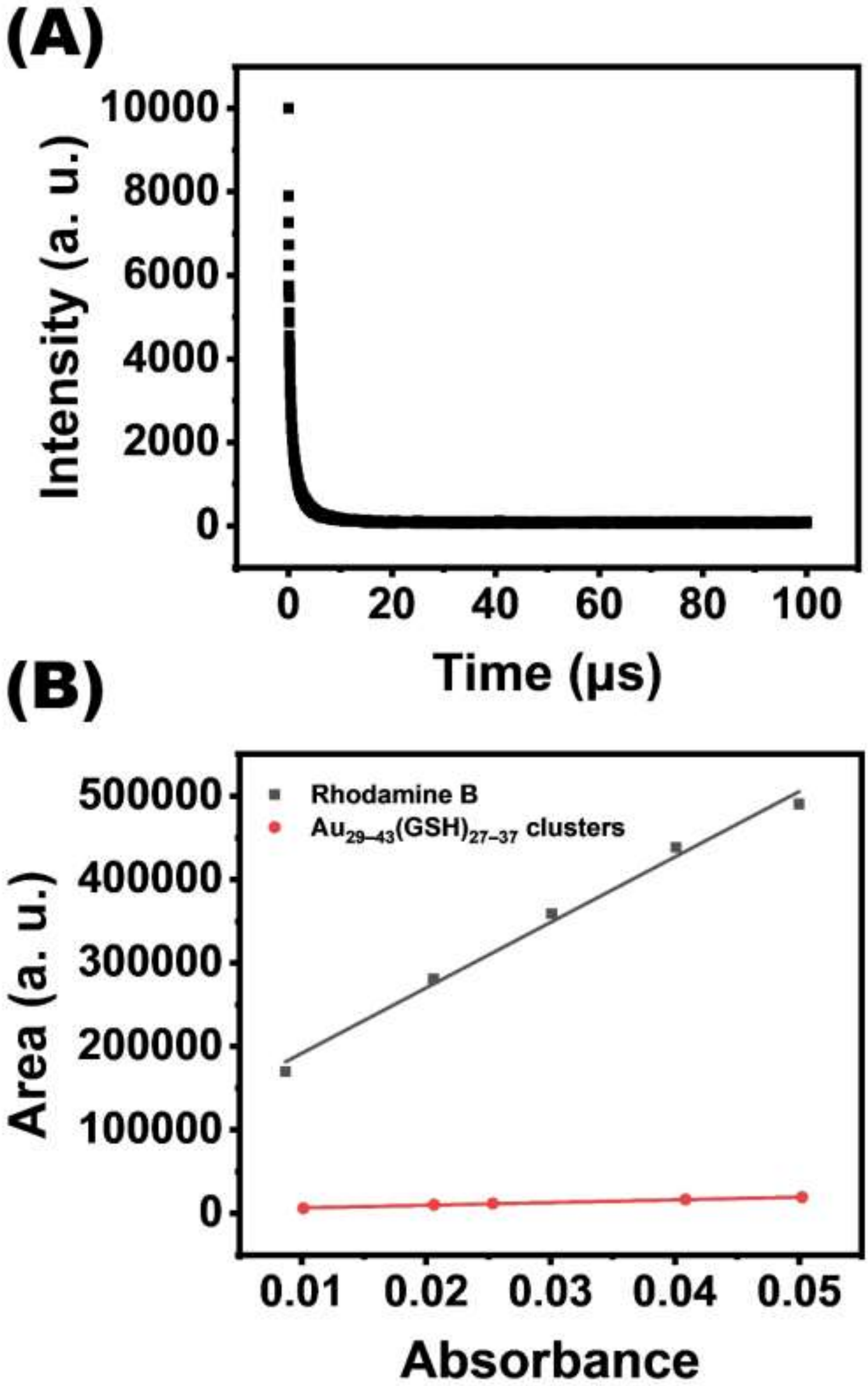

Figure S3. (A) time-resolved luminescence decay, and (B) quantum yield measurement of the $Au_{29-43}(GSH)_{27-37}$ clusters.

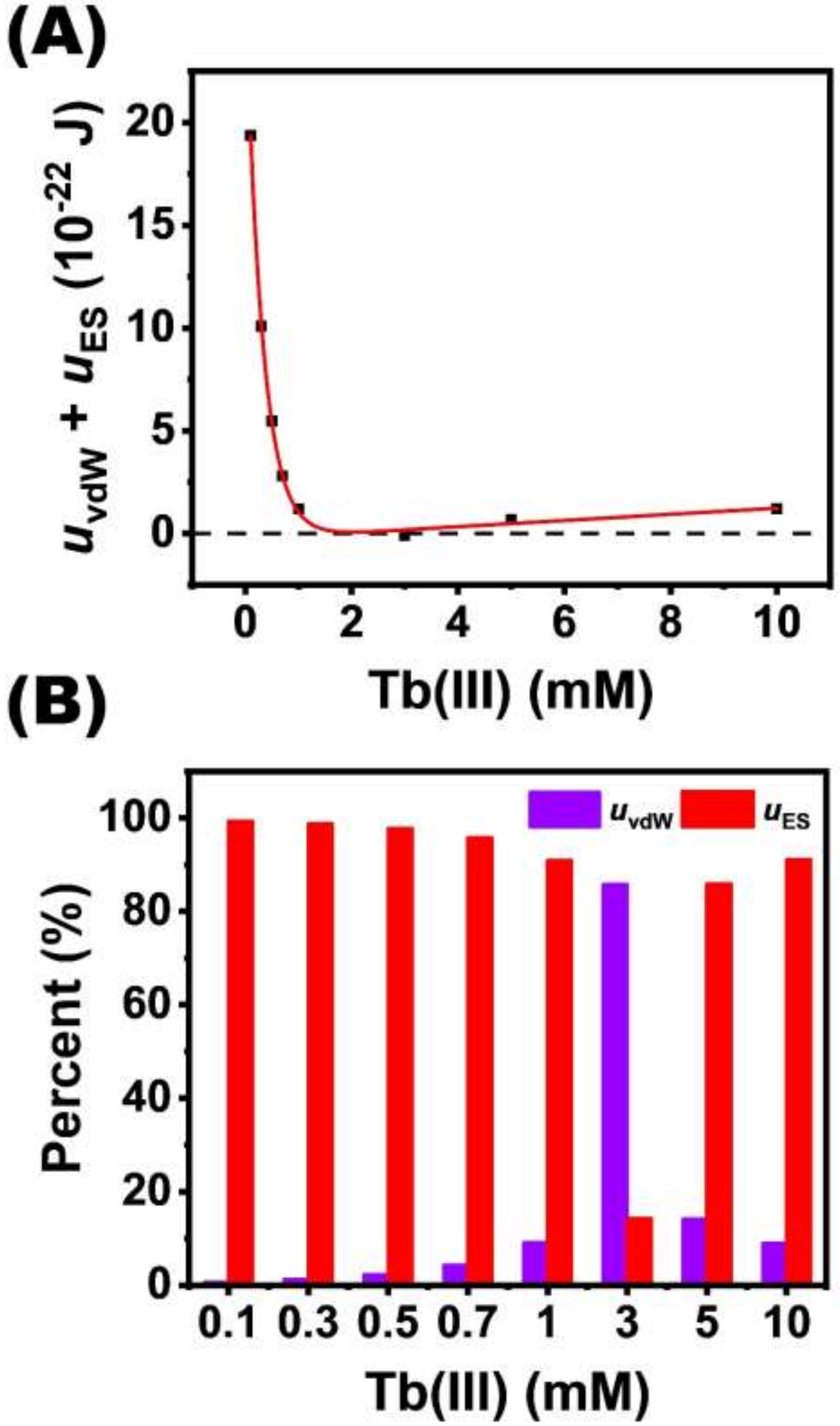

Figure S4. (A) Plot of calculated total interaction energy ($u_{total} = u_{vdW} + u_{ES}$) versus Tb^{3+} concentration. (B) Relative contributions of van der Waals attraction (u_{vdW}) and electrostatic repulsion (u_{ES}) to the u_{total} values at varying Tb^{3+} concentrations.

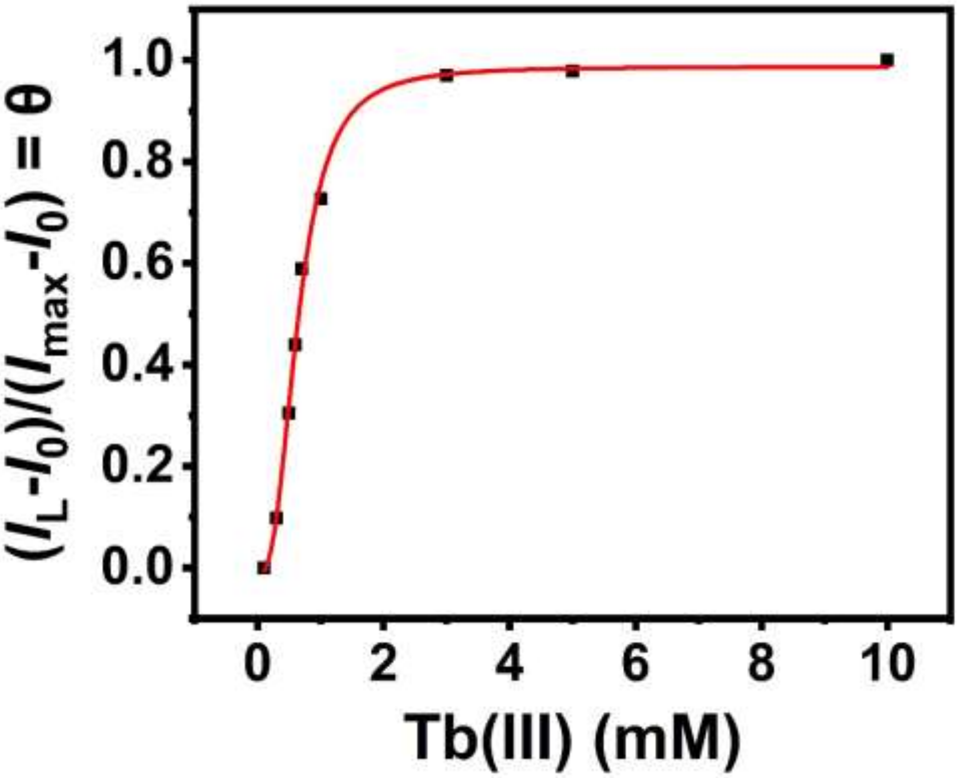

Figure S5. Binding isotherm for determination of the dissociation constant (K_d) of Tb^{3+} binding to $Au_{29-43}(GSH)_{27-37}$ clusters using the Hill equation. Plot of θ as a function of Tb^{3+} concentration. I_0, I_{max} and I_L correspond to the luminescence intensity of the clusters at the lowest Tb^{3+} concentration (comparable to the blank solution), at maximum saturation concentration, and at various intermediate Tb^{3+} concentrations, respectively.

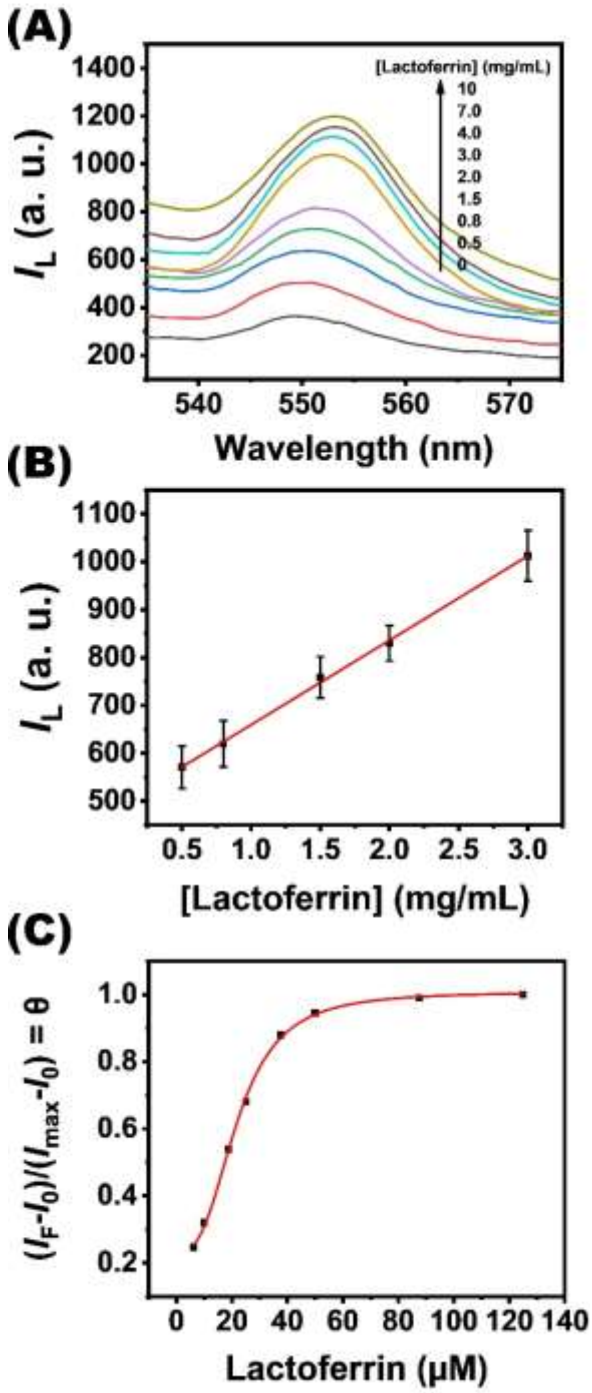

Figure S6. (A) Luminescence spectra of the Tb^{3+}–Lf complexes at different Lf concentrations in 50 mM HEPES/3.75 mM $NaHCO_3$ buffer (pH 7.0). Luminescence spectra was recorded at an excitation wavelength of 298 nm in the presence of 1000 µM Tb^{3+}. (B) Calibration curve of luminescence intensity versus Lf concentration with a linear range of 0.5–3.0 mg/mL. (C) Binding isotherm for determination of the binding constant (K_b) of Lf binding to Tb^{3+} using the Hill equation. Plot of θ as a function of Lf concentration.

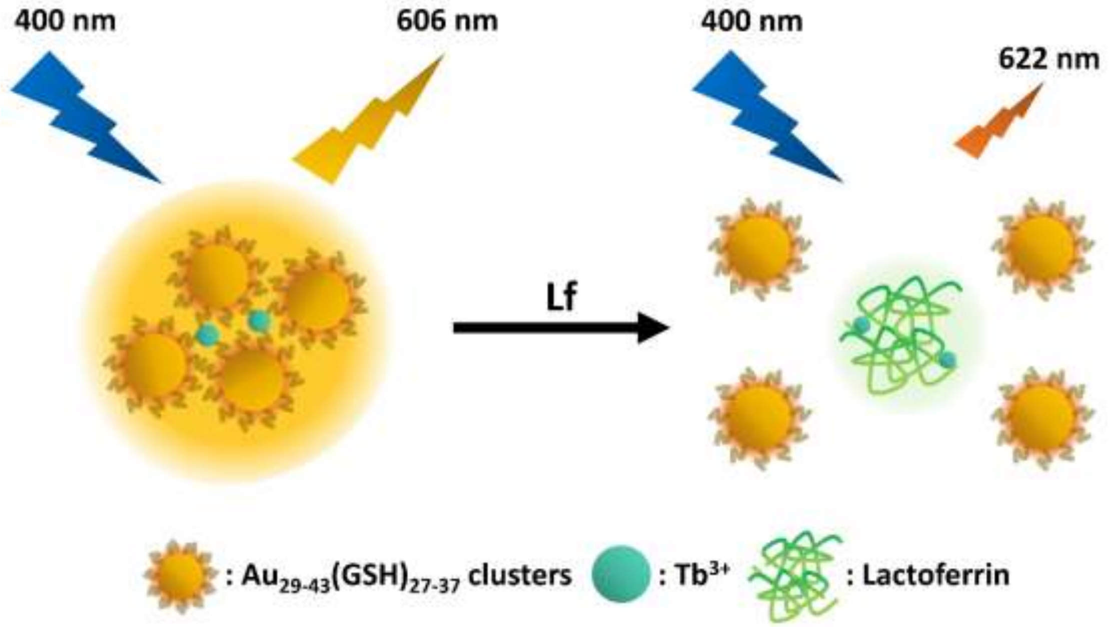

Figure S7. Schematic illustration of the Lf-mediated disassembly of Tb^{3+}–$Au_{29-43}(GSH)_{27-37}$ aggregates into a dispersed state.

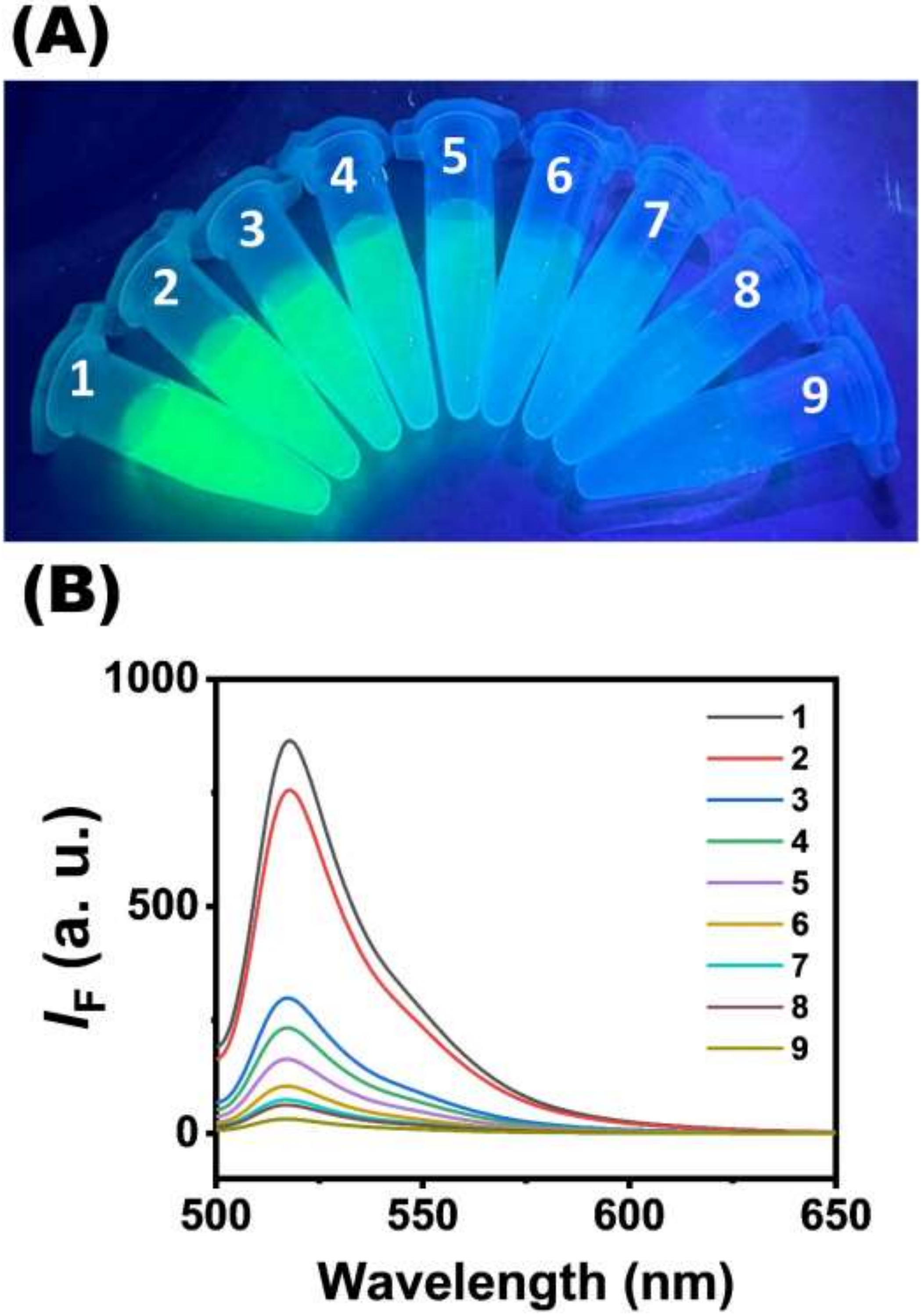

Figure S8. (A) Photograph of the filtrates collected after spin column purification of the BDP-FL–conjugated clusters under UV light excitation. (B) Fluorescence spectra of the filtrates corresponding to purification cycles 1–9. Labels 1–9 correspond to successive purification cycles

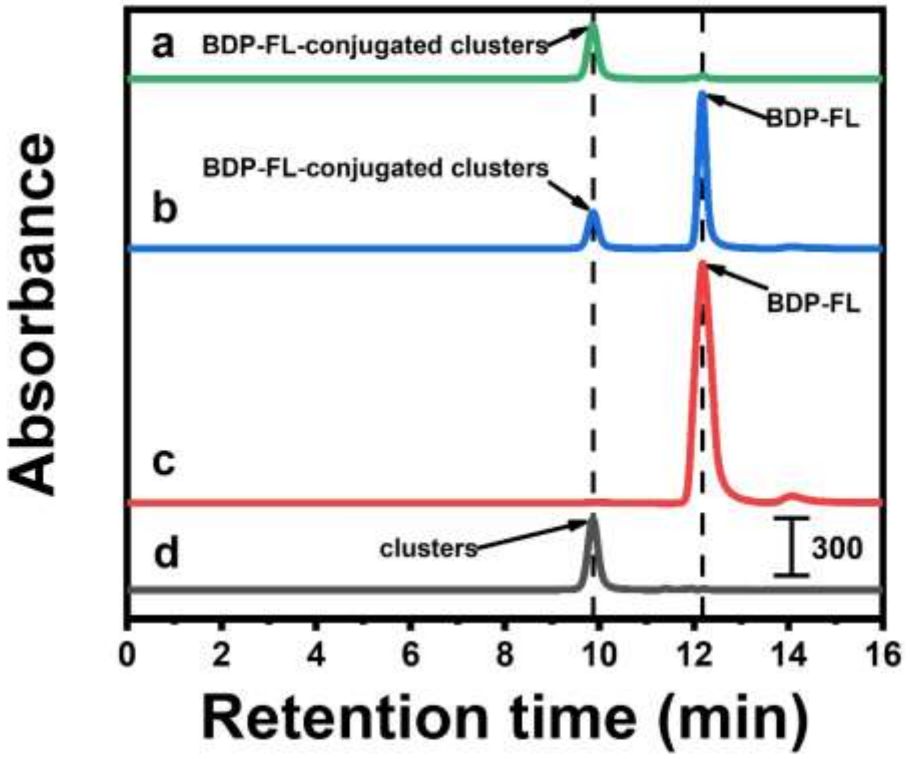

Figure S9. Gel permeation chromatograms of (a) the purified BDP-FL-conjugated $Au_{29-43}(GSH)_{27-37}$ clusters, (b) the unpurified BDP-FL-conjugated $Au_{29-43}(GSH)_{27-37}$ clusters, (c) BDP-FL NHS ester, and (d) the $Au_{29-43}(GSH)_{27-37}$ clusters. The mobile phase is 150 mM phosphate buffer (pH 7.0).

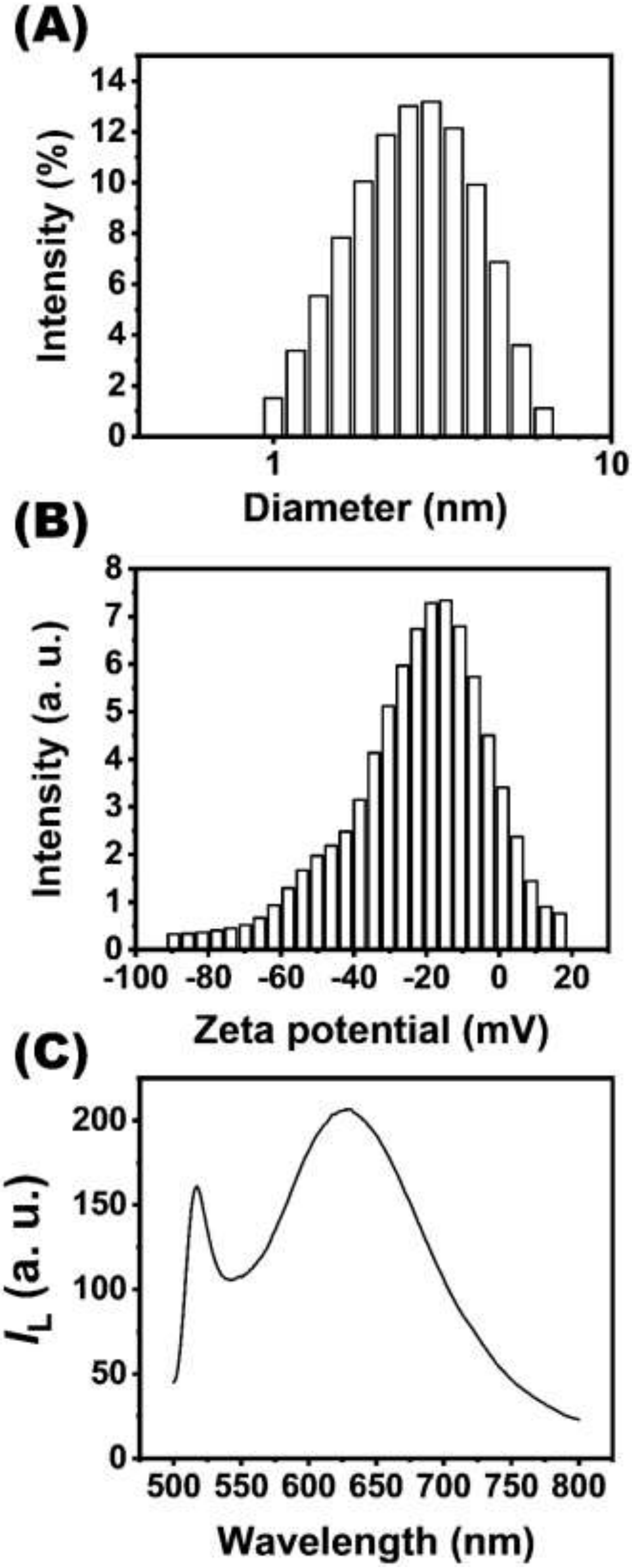

Figure S10. (A) Hydrodynamic diameter, (B) zeta potential, and (C) luminescence spectra of the purified BDP-FL-conjugated $Au_{29-43}(GSH)_{27-37}$ clusters.

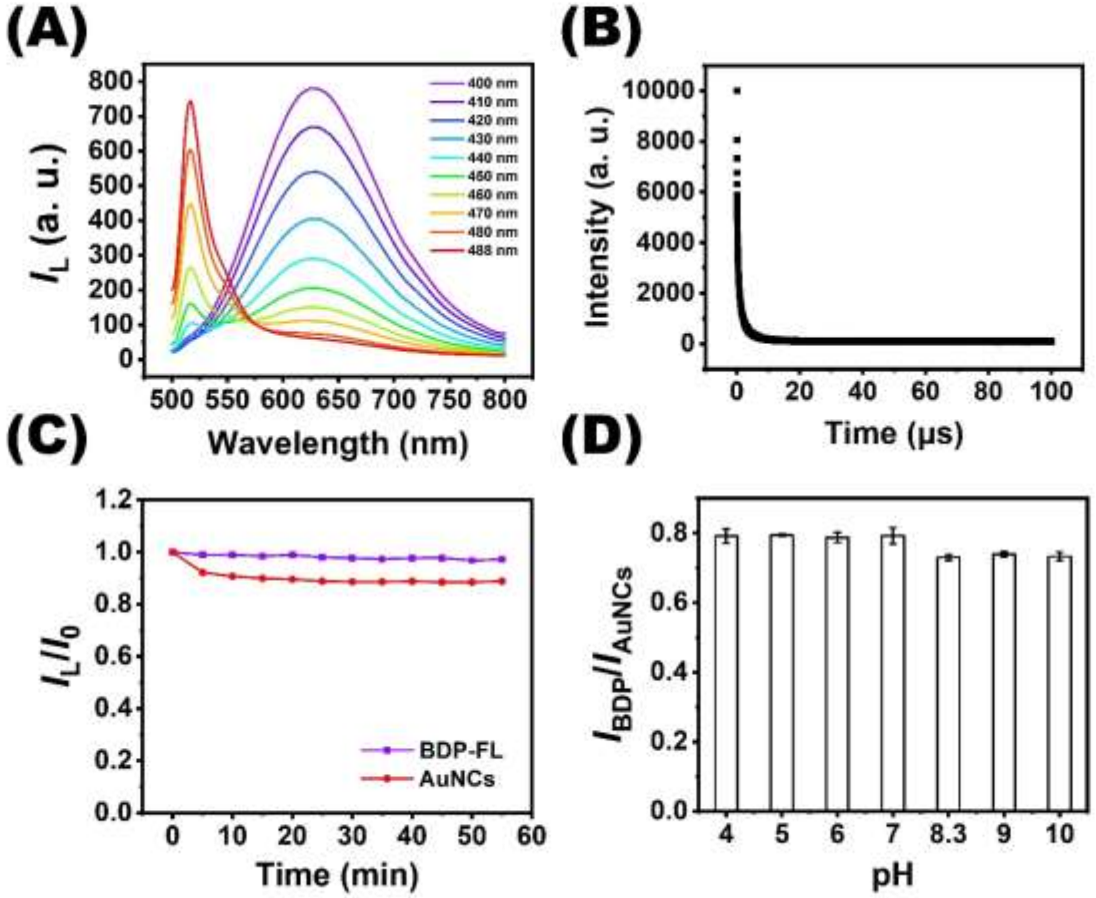

Figure S11. (A) Luminescence spectra of the BDP-FL-conjugated $Au_{29-43}(GSH)_{27-37}$ clusters under different excitation wavelengths in 10 mM HEPES buffer (pH 7.0). (B) Time-resolved luminescence decay of the $Au_{29-43}(GSH)_{27-37}$ clusters in the BDP-FL-conjugated $Au_{29-43}(GSH)_{27-37}$ clusters. (C) Photostability of BDP-FL molecules and the $Au_{29-43}(GSH)_{27-37}$ clusters in the BDP-FL-conjugated $Au_{29-43}(GSH)_{27-37}$ clusters under continuous 450-nm irradiation. (D) The I_{BDP}/I_{AuNCs} ratios of BDP-FL-conjugated $Au_{29-43}(GSH)_{27-37}$ clusters over a pH range of 4.0–10.0 in 10 mM HEPES buffer.

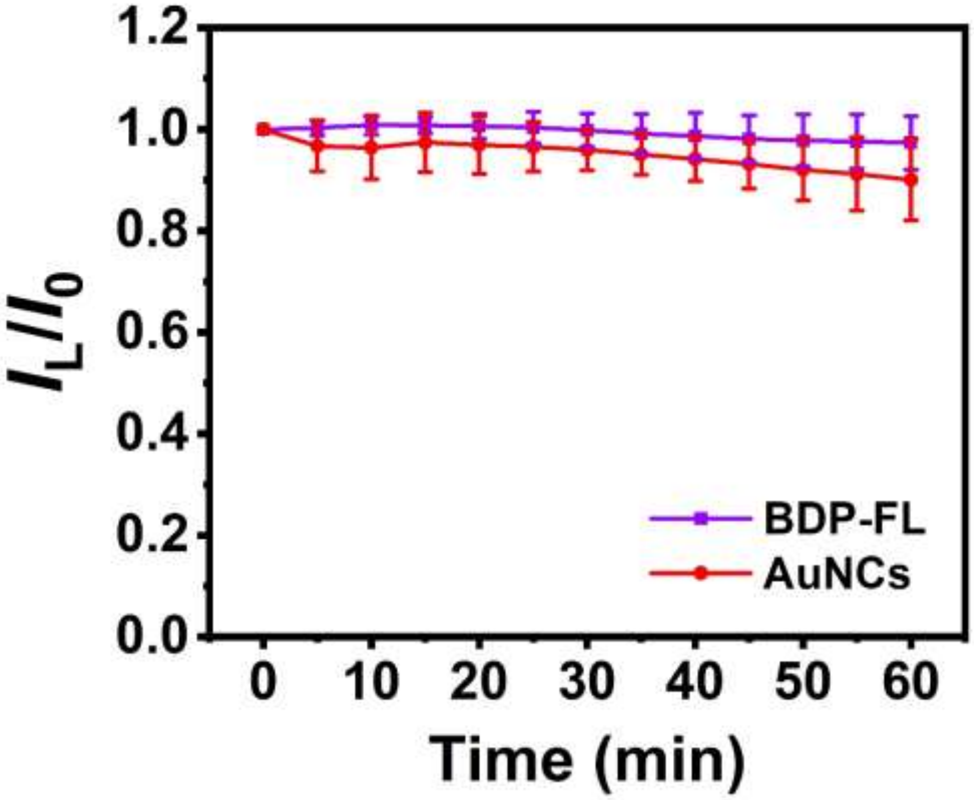

Figure S12. Photostability of BDP-FL molecules and the $Au_{29-43}(GSH)_{27-37}$ clusters in the BDP-FL-conjugated $Au_{29-43}(GSH)_{27-37}$ clusters with the addition of 3000 μM Tb^{3+} under continuous 488-nm irradiation.

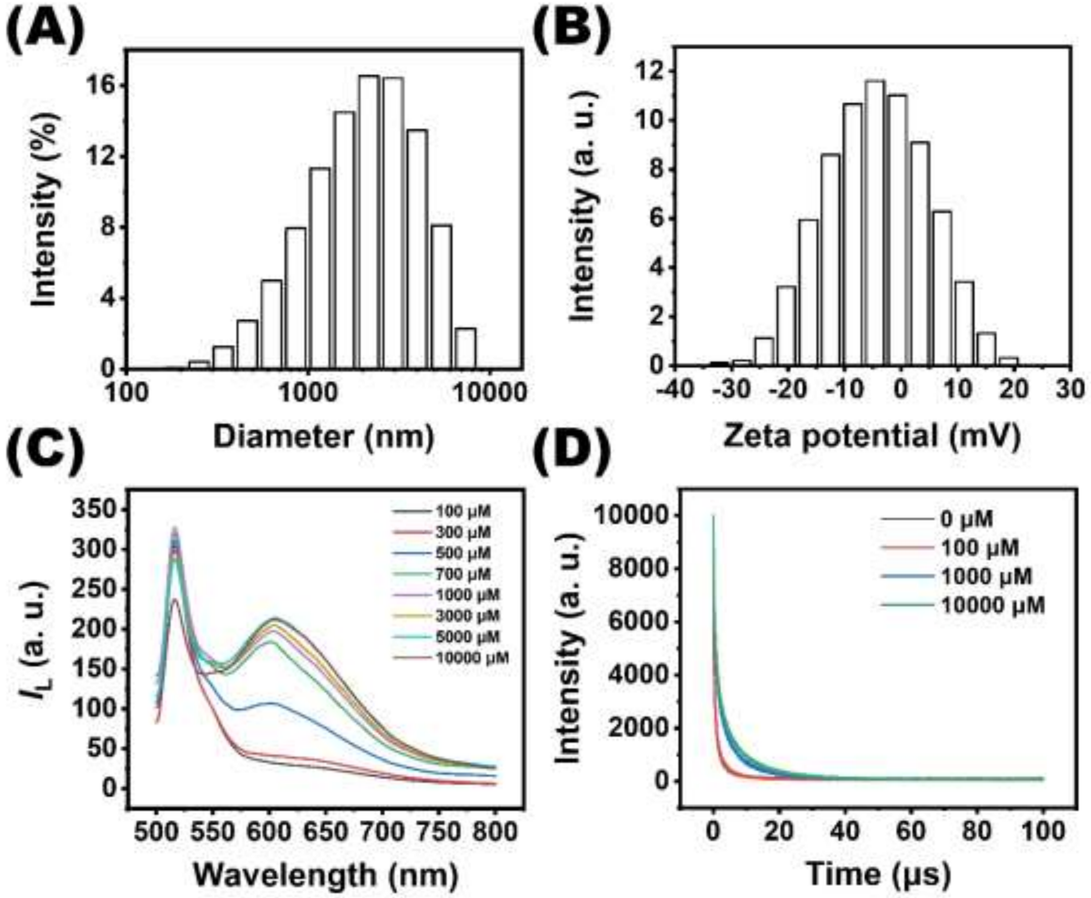

Figure S13. (A) Hydrodynamic diameter and (B) zeta potential of BDP-FL-conjugated AIEE dots formed in the presence of 3000 μM Tb^{3+}. (C) Luminescence spectra and (D) time-resolved luminescence decay of the BDP-FL-conjugated AIEE dots formed at Tb^{3+} concentrations. The buffer solution is 10 mM HEPES (pH 7.0). The excitation wavelength is set to 488 nm.

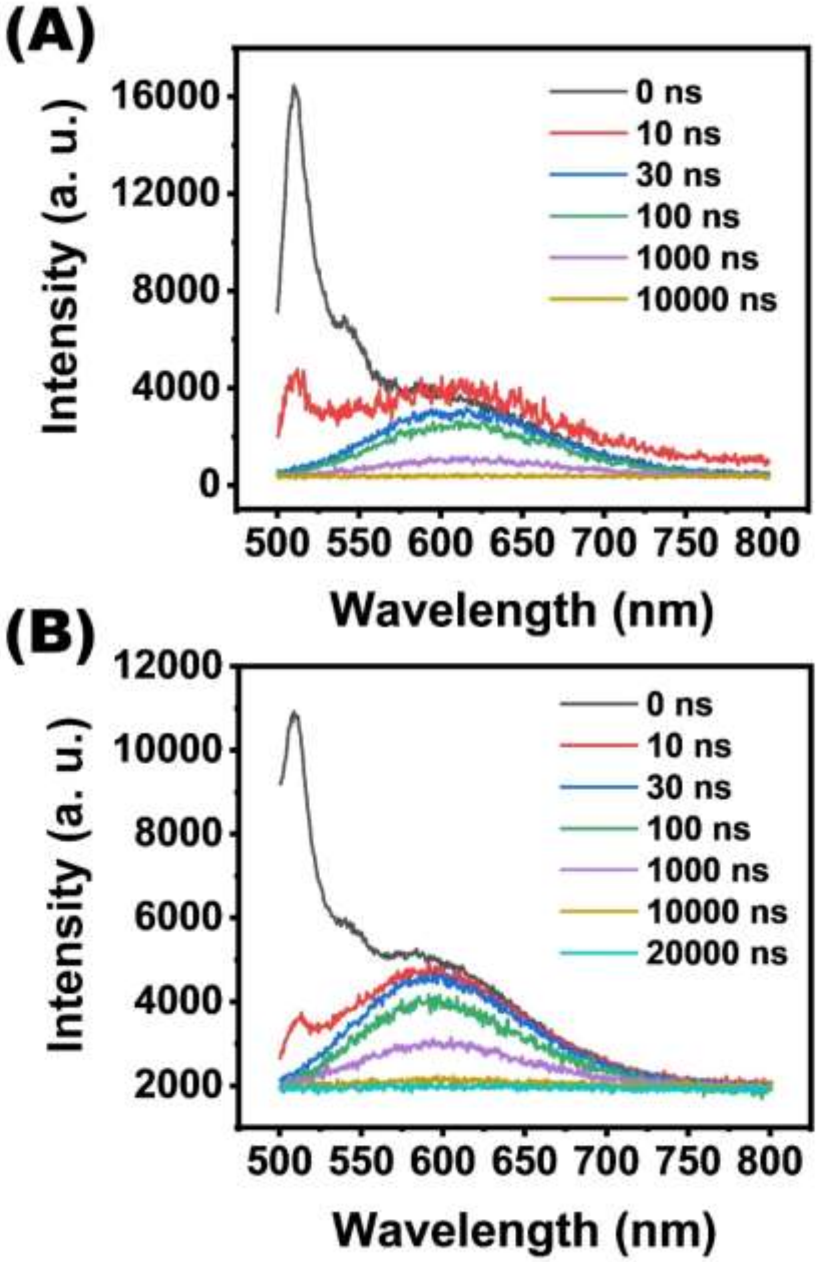

Figure S14. Time-resolved luminescence spectra of the BDP-FL-conjugated cluster in the (A) absence and (B) presence of 0.01 M Tb^{3+}. The buffer solution is 10 mM HEPES (pH 7.0).

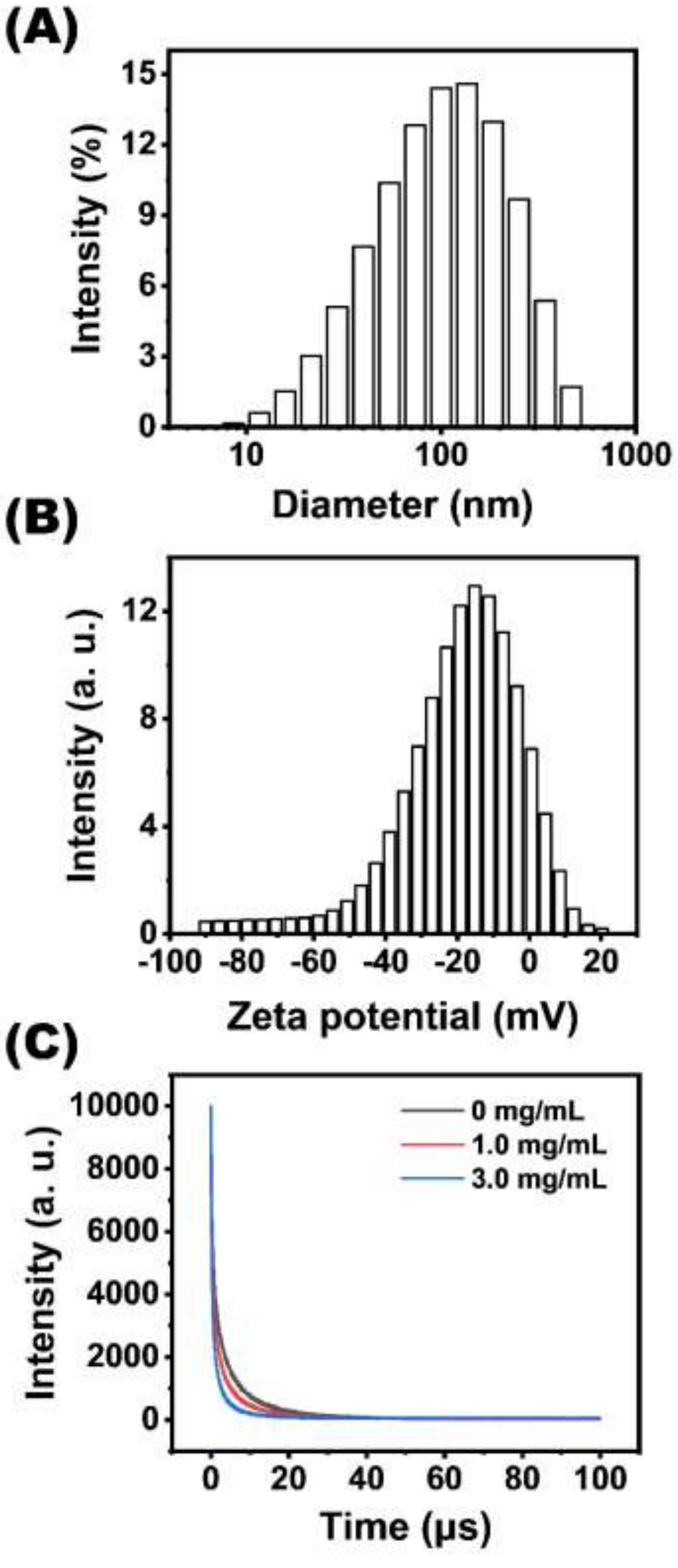

Figure S15. (A) Hydrodynamic diameter, (B) zeta potential, and (C) luminescence lifetimes of the AuNCs in the BDP-FL-conjugated AIEE dots after incubation with (A, B) 3.0 mg/mL Lf and (C) different Lf concentrations.

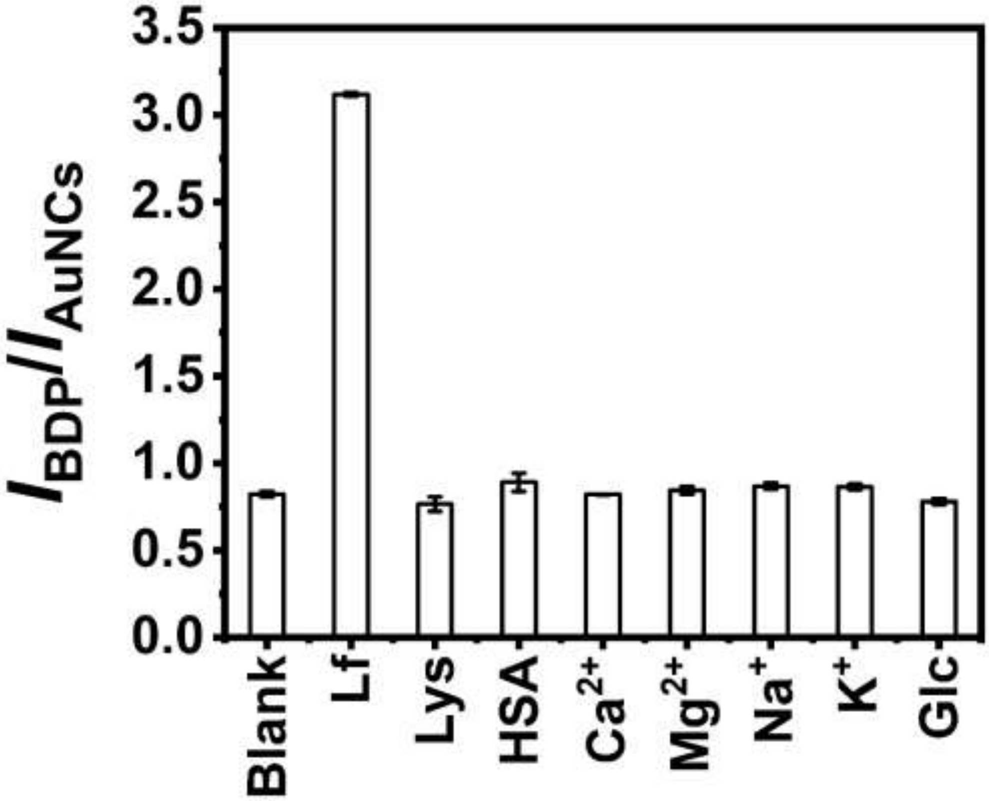

Figure S16. Selectivity of the BDP-FL–conjugated AIEE dots in the presence of mimicking tear matrix components, including 2.11 mg/mL Lf, 1.89 mg/mL Lys, 0.021 mg/mL HSA, 2.0 mM Ca^{2+}, 1.1 mM Mg^{2+}, 170 mM Na^{+}, 42 mM K^{+}, and 0.029 mg/mL glucose (Glc). The excitation wavelength is set to 488 nm.

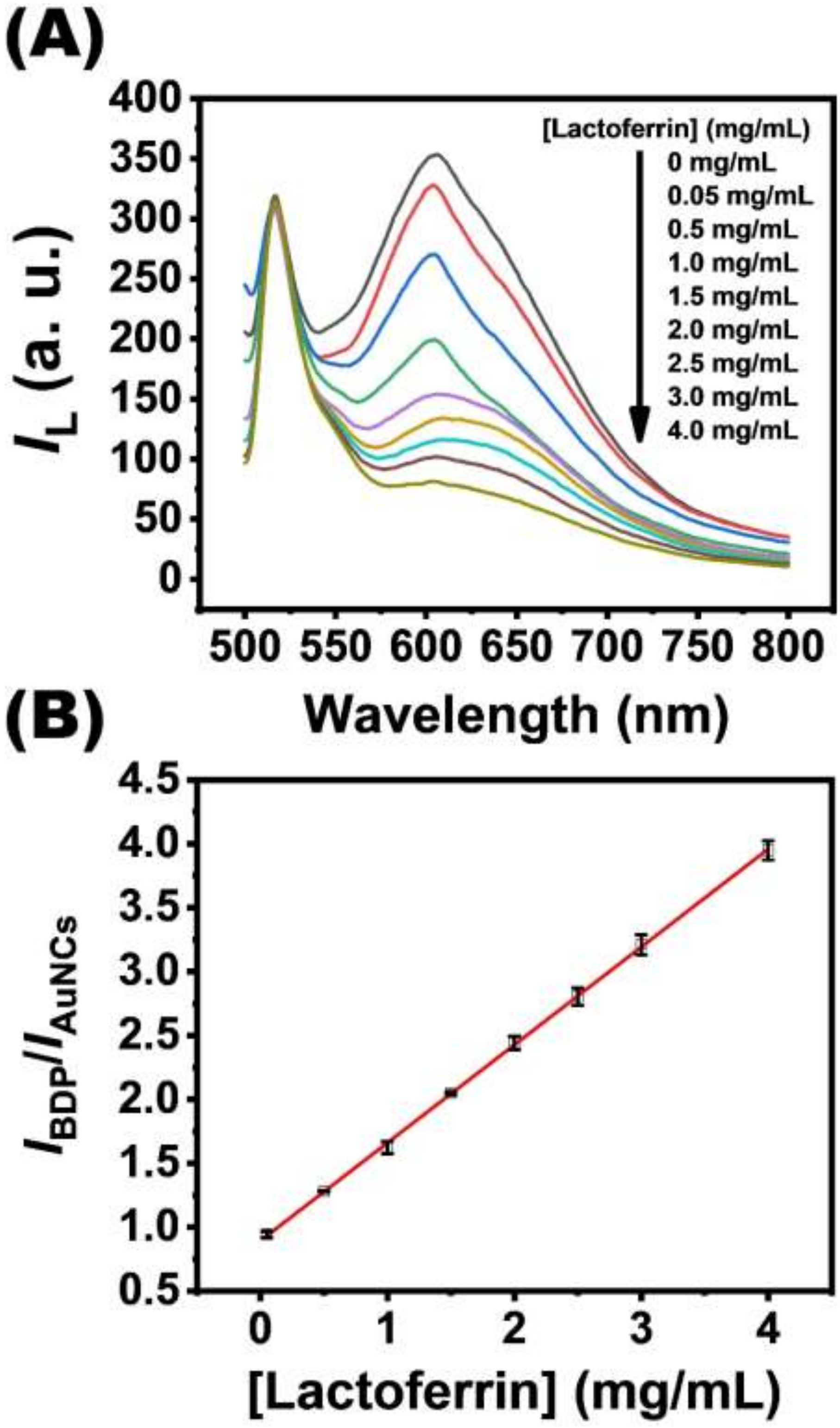

Figure S17. (A) Luminescence spectra of BDP-FL–conjugated AIEE dots incubated with tear samples spiked with varying concentrations of standard Lf (0.05–4.0 mg/mL). (B) Corresponding calibration curve of the I_{BDP}/I_{AuNCs} ratio versus the spiked concentration of Lf. The excitation wavelength is set to 488 nm.

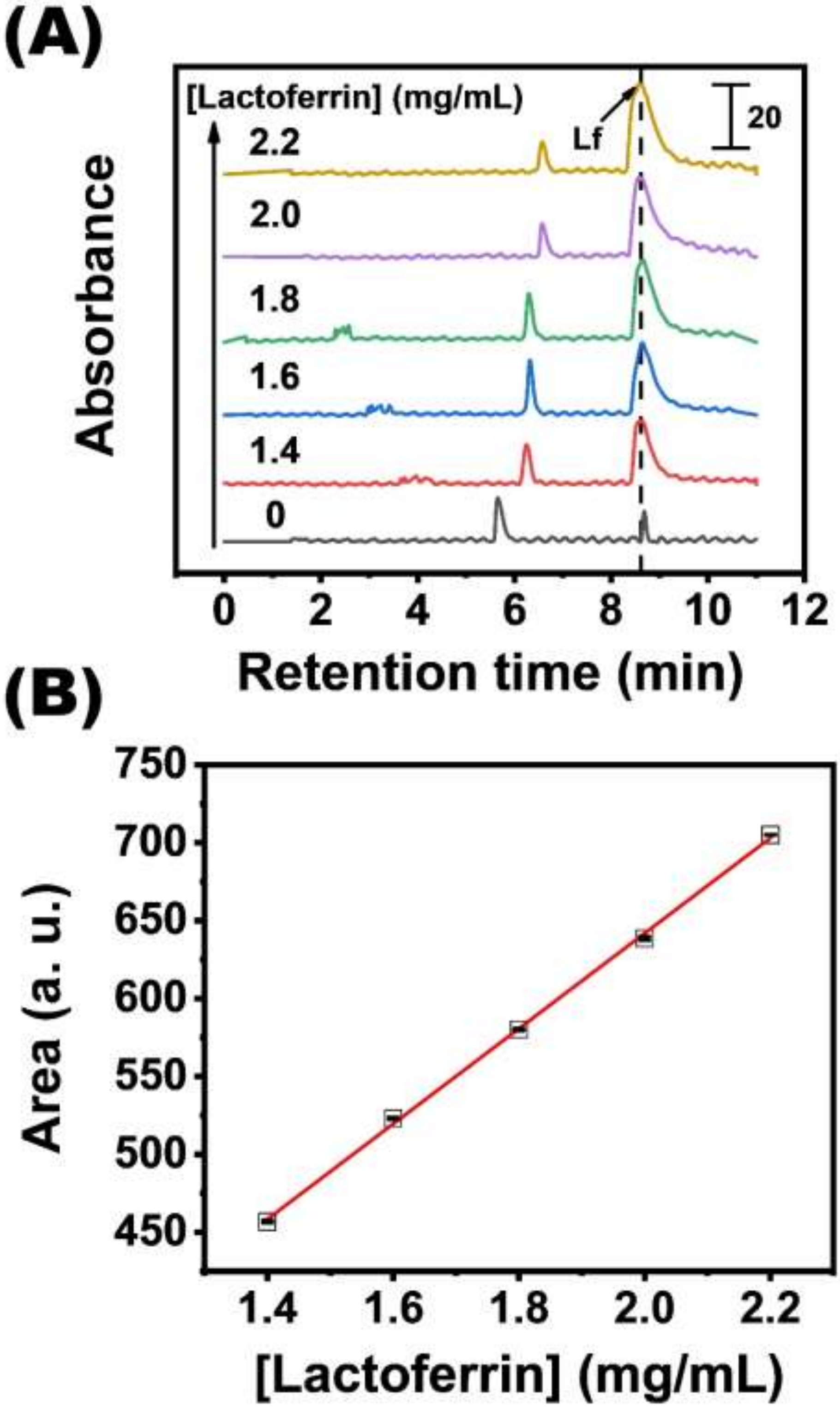

Figure S18. (A) Electropherograms of tear samples spiked with 0, 1.4, 1.6, 1.8, 2.0, and 2.2 mg/mL Lf. The background electrolyte was 50 mM phosphate buffer containing 6.0 M urea (pH 4.0). (B) Calibration plot of the peak area of Lf versus its spiked concentration.

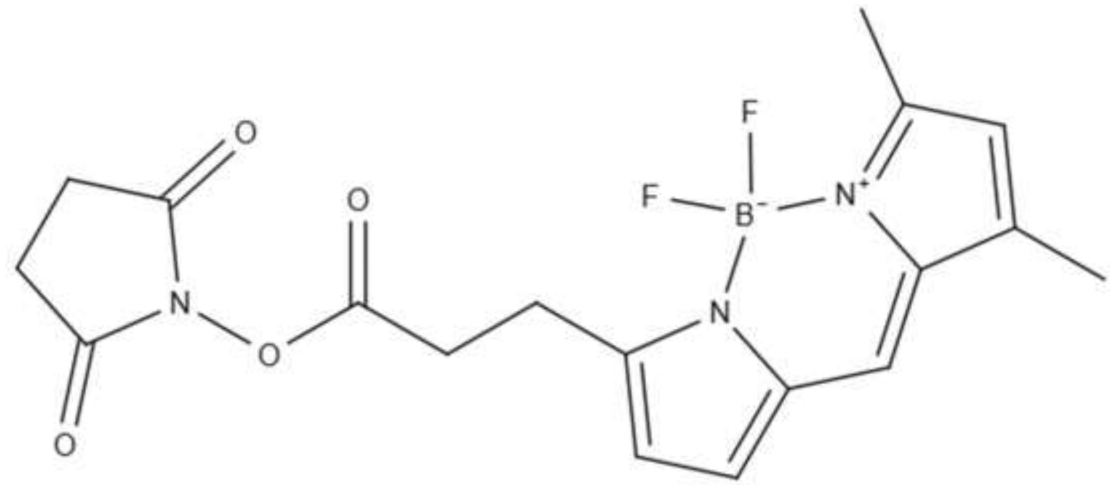

Figure S19. Chemical structure of BDP-FL NHS ester

Table S1. Calculated ionic strength and Debye screening length (κ^{-1}) of the $Au_{29-43}(GSH)_{27-37}$ cluster system in 10 mM HEPES buffer (pH 7.0) at different Tb^{3+} concentrations.

**[Tb^{3+}] (μM)**	**Ionic strength (M)**	**Debye length (m)**
100	2.46×10^{-3}	6.13×10^{-9}
300	2.58×10^{-3}	5.98×10^{-9}
500	2.70×10^{-3}	5.85×10^{-9}
700	2.82×10^{-3}	5.72×10^{-9}
1000	3.00×10^{-3}	5.55×10^{-9}
3000	4.20×10^{-3}	4.69×10^{-9}
5000	5.40×10^{-3}	4.14×10^{-9}
10000	8.40×10^{-3}	3.32×10^{-9}

Table S2. Zeta potential (V), calculated surface potential (ϕ_S), and electrostatic interaction energy (u_{ES}) values of the $Au_{29-43}(GSH)_{27-37}$ clusters as a function of Tb^{3+} concentration.

**[Tb^{3+}] (μM)**	**Zeta potential (V)**	**Surface potential (ϕ_S)**	**u_{ES} (J)**
100	-4.61×10^{-2}	-6.18×10^{-2}	1.95×10^{-21}
300	-3.68×10^{-2}	-4.43×10^{-2}	1.02×10^{-21}
500	-2.85×10^{-2}	-3.27×10^{-2}	5.60×10^{-22}
700	-2.12×10^{-2}	-2.37×10^{-2}	2.94×10^{-22}
1000	-1.44×10^{-2}	-1.59×10^{-2}	1.31×10^{-22}
3000	1.92×10^{-3}	2.14×10^{-3}	2.18×10^{-24}
5000	1.18×10^{-2}	1.35×10^{-2}	7.99×10^{-23}
10000	1.58×10^{-2}	1.88×10^{-2}	1.34×10^{-22}

Table S3. Van der Waals interaction energy (u_{vdW}), electrostatic interaction energy (u_{ES}), and bridging interaction energy (u_B) values and their relative contributions of the $Au_{29-43}(GSH)_{27-37}$ clusters as a function of Tb^{3+} concentration.

**[Tb^{3+}] (μM)**	**u_{vdW} (J)**	**u_{ES} (J)**	**u_B (J)**
100	-1.31×10^{-23} (0.57%)	1.95×10^{-21} (84.6%)	-3.42×10^{-22} (14.9%)
300	-1.31×10^{-23} (0.95%)	1.02×10^{-21} (74.2%)	-3.42×10^{-22} (24.9%)
500	-1.31×10^{-23} (1.43%)	5.60×10^{-22} (61.2%)	-3.42×10^{-22} (37.4%)
700	-1.31×10^{-23} (2.02%)	2.94×10^{-22} (45.3%)	-3.42×10^{-22} (52.7%)
1000	-1.31×10^{-23} (2.69%)	1.31×10^{-22} (27.0%)	-3.42×10^{-22} (70.3%)
3000	-1.31×10^{-23} (3.66%)	2.18×10^{-24} (0.61%)	-3.42×10^{-22} (95.7%)
5000	-1.31×10^{-23} (3.01%)	7.99×10^{-23} (18.4%)	-3.42×10^{-22} (78.6%)
10000	-1.31×10^{-23} (2.68%)	1.34×10^{-22} (27.3%)	-3.42×10^{-22} (70.0%)

Table S4. Comparison of the analytical performance of the BDP-FL–conjugated AIEE dots for the detection of Lf with previously reported fluorescent and luminescent sensors.

Materials[a]	Detection mode[b]	linear range (mg/mL)	LOD (μg/mL)	Reference
Lf aptamer-conjugated CDs and GONSs	FRET	4 to 16	2.48	ACS Omega. 2022 10, 37964–37970
Lf aptamer-conjugated CDs and GONSs	Fluorescence polarization	0.66 to 3.32	1.4	Sci. Rep. 2023, 13, 15179
$TbCl_3$	Turn-on fluorescence	Not given	Not given	Sci Rep 2024, 14, 14505.
$TbCl_3$ and microfluidic paper	Turn-on fluorescence	0 to 4	100	ACS Appl. Mater. Interfaces 2015, 7, 44, 24864–24875
$TbCl_3$ and lateral flow strip	Turn-on fluorescence	0 to 5	570	Sens. Actuators, B 2023, 378, 133128.
GODs and MnO_2 nanosheets	Turn-on fluorescence	5×10^{-6} to 1.6×10^{-3}	1.7×10^{-3}	Inorg. Chem. Commun. 2022, 143, 109751.
Carboxyl-rich CDs	Turn-off fluorescence	0 to 6.62×10^{-3}	0.776	Polymers 2021, 13, 4317
FITC-conjugated aptamer and aptamer-capped AgNPs	Fluorescence polarization	0.2×10^{-6} to 25×10^{-3}	0.1×10^{-3}	Anal. Chem. 2017, 89, 5900–5908
ZnO nanoflower-coated MIP	Turn-off fluorescence	0.0625 to 1.00	20.7	Food. Chem, 2025 489, 145036.
BDP-modified AuNCs and Tb^{3+}	Ratiometric detection	0.01 to 4.0	3.4	This work

[a]CDs, carbon dots; GONSs, graphene oxide nanosheets; GQDs, graphene quantum dots; AgNPs, silver nanoparticles; MIP, molecularly imprinted polymer.

[b]FRET, fluorescence resonance energy transfer

Table S5. Recovery and relative standard deviation (RSD) values for lactoferrin determination in spiked tear samples using the BDP-FL–conjugated AIEE dots.

**Spiked LF (mg/mL)**	**Found LF (mg/mL)**	**Recovery (%)**	**RSD (n = 3) (%)**
0.05	0.051	101.8	2.57
0.50	0.500	99.98	0.38
1.50	1.52	101.0	0.59
2.50	2.52	100.6	2.44
